# An Experimental Analysis on Multicepstral Projection Representation Strategies for Dysphonia Detection

**DOI:** 10.3390/s23115196

**Published:** 2023-05-30

**Authors:** Rodrigo Colnago Contreras, Monique Simplicio Viana, Everthon Silva Fonseca, Francisco Lledo dos Santos, Rodrigo Bruno Zanin, Rodrigo Capobianco Guido

**Affiliations:** 1 Department of Computer Science and Statistics, Institute of Biosciences, Letters and Exact Sciences, São Paulo State University, São José do Rio Preto 15054-000, SP, Brazil; guido@ieee.org; 2 Federal Institute of São Paulo, São José do Rio Preto 15030-070, SP, Brazil; monique.viana03@gmail.com (M.S.V.); everthon@ifsp.edu.br (E.S.F.); 3 Faculty of Architecture and Engineering, Mato Grosso State University, Cáceres 78217-900, MT, Brazil; franciscolledo@unemat.br (F.L.d.S.); rodrigo.zanin@unemat.br (R.B.Z.)

**Keywords:** dysphonia detection, voice disorder detection, pattern recognition, cepstral analysis, machine learning

## Abstract

Biometrics-based authentication has become the most well-established form of user recognition in systems that demand a certain level of security. For example, the most commonplace social activities stand out, such as access to the work environment or to one’s own bank account. Among all biometrics, voice receives special attention due to factors such as ease of collection, the low cost of reading devices, and the high quantity of literature and software packages available for use. However, these biometrics may have the ability to represent the individual impaired by the phenomenon known as dysphonia, which consists of a change in the sound signal due to some disease that acts on the vocal apparatus. As a consequence, for example, a user with the flu may not be properly authenticated by the recognition system. Therefore, it is important that automatic voice dysphonia detection techniques be developed. In this work, we propose a new framework based on the representation of the voice signal by the multiple projection of cepstral coefficients to promote the detection of dysphonic alterations in the voice through machine learning techniques. Most of the best-known cepstral coefficient extraction techniques in the literature are mapped and analyzed separately and together with measures related to the fundamental frequency of the voice signal, and its representation capacity is evaluated on three classifiers. Finally, the experiments on a subset of the Saarbruecken Voice Database prove the effectiveness of the proposed material in detecting the presence of dysphonia in the voice.

## 1. Introduction

User authentication systems can be defined by objects that an individual has, such as an access card; or records, such as login data [1]. Despite being successfully used in many applications, these systems have weaknesses in their operation, since the user may lose the access object or forget the information necessary for authentication. Alternatively, biometric authentication systems (BASs) [2] conduct user recognition based on their physiological and behavioral properties, which are known as biometrics [3], and hence are not trivially lost or forgotten. Voice, for example, is a biometric with great potential for applications in BASs [4], which defines voice-based authentication systems (VASs) [5]. Among the advantages of its use, we can highlight its uniqueness with the user, since each individual has a voice characterized by their unique vocal tract [6]; its ease of collection [7], since the biometrics reading is performed in a completely noninvasive way and without any physical interaction with devices, which also guarantees asepsis; a large quantity of literature [8] and computational packages [9] available for use; and the low cost of devices [10] used for collection, since any electronic system connected to a microphone can be used for this purpose [11]. For example, we can mention cell phones, which, due to their recent cheapening, are used by a large part of the world’s population [12], and consequently, can be used in voice recognition and authentication [13]. In addition, smart assistance devices that respond to voice commands from registered users such as Amazon Alexa${}^{\mathrm{TM}}$ [14], Google Home${}^{\mathrm{TM}}$ [15], and Apple Siri${}^{\mathrm{TM}}$ [16], among others [17], are becoming popular.

Associated with the voice, there is a clinical phenomenon known as dysphonia [18,19,20], which corresponds to changes in the voice of individuals caused by some disease that disturbs the human vocal tract. As an example, we can highlight very common diseases, such as colds and flu [21], and more complex diseases such as Parkinson’s [22,23] and Alzheimer’s [24]. Specifically, according to Hur et al. [25], nearly 8% of the US adult population is affected by some form of dysphonia. In addition to all the physiological damage and the decrease in quality of life [26] that this phenomenon can cause, it is also known that dysphonia can compromise the performance of voice-based recognition systems. Rohlfing et al. [27], for example, found that the command recognition performance presented by Google Voice${}^{\mathrm{TM}}$ was reduced from 93.8% to 68.7% when individuals with voice dysphonia started to use the system, which corresponds to a reduction of more than 1/4 in accuracy. To get around this adversity, automatic detection methods for voice dysphonia have been proposed in the literature [28], since the prior detection of dysphonia can be useful in the sense that the system makes use, for example, of specific routines for filtering to improve recognition of an affected speaker. Among such methods, the most common are those that make use of strategies based on machine learning [29,30] and analysis of handcrafted features [31,32], which are obtained from the voice signal, the most common in the subject being those defined by mappings and perturbations of the fundamental frequency ($F_{0}$) of the signal [33,34] and those based on cepstral coefficients (CCs) [35] such as Mel-frequency cepstral coefficients (MFCC) [36] and linear prediction cepstral coefficients (LPCC) [37], among others [38,39]. In most works that use CCs to detect voice dysphonia, only one cepstral technique is considered to compose the sound signal representation model, and there are few works in which more than one type of cepstral feature is used. Furthermore, CCs are originally defined by a matrix $M\in R^{n_{\mathrm{ceps}}\times n_{\mathrm{frames}}}$, with $n_{\mathrm{ceps}}$ being the number of CCs and $n_{\mathrm{frames}}$ the number of temporal windows considered in the signal splitting, which must be calculated for each sample that we intend to represent, which can result in a high memory cost in the developed model. To get around this adversity, a commonly used strategy consists of projecting this matrix through some statistical mapping, such as sum or average on the frame axis. However, to the best of our knowledge, there is no work that demonstrates the efficiency of certain mappings on the CCs, especially when considered together, in the representation of a voice signal in the dysphonia detection problem.

In this work, a new framework based on the representation of a voice signal by the multiprojection of sets of CCs is proposed in order to detect the presence of dysphonia in the speech signal. In detail, massive experimentation is conducted on a set of eight CC extraction techniques, in which these features are analyzed in isolation and together, and their ability to represent the speech signal is evaluated against well-established classification techniques. In addition, the classification performance of the proposed framework is also analyzed considering the use of traditional features of voice signal representation such as those based on the Fundamental Frequency. Finally, results are obtained from a subset of the Saarbruecken Voice Database [40] with specific voice signals of individuals affected by dysphonia. In this way, the main scientific advances contained in this work can be summarized in the following:

- A new framework for detecting dysphonia in voice signals;
- The formalization of a process involving the fusion and multiple projections of CCs to represent the voice signal;
- An extensive number of experimental results were conducted on the Saarbruecken Voice Database with respect to diverse configurations involving various techniques of extracting CCs and other measurements of the speech signal, such as fundamental frequency measurements.

The remainder of the text is divided as follows: Section 2 contains a detailed review of related works on the topic of voice dysphonia detection using artificial intelligence, providing an overview of the state of the art of the subject; in Section 3, a tutorial review of the general procedure for extracting CCs in their matrix form is carried out; in Section 4, the methodology adopted in this study is presented, emphasizing our contributions and how they are validated; in Section 5, a generalization for the vector representation of CCs through mappings is proposed, in addition to a multistep framework specialized in improving the representation of voice signals for the problem; in Section 6, practical instances of the proposed generalizations are given; the results that demonstrate the effectiveness of the proposed material are presented in Section 7, offering a careful analysis of the data collected and the conclusions obtained; the work concludes with final considerations and suggestions for future developments in Section 8.

## 2. Related Works

Dysphonia, which is the phenomenon analyzed in this work, is a disorder in the human voice caused by a morphological and functional disturbance of the pneumophonoarticulatory apparatus [41]. It is possible to analyze and detect this type of disorder through clinical procedures such as laryngoscopy and laryngeal stroboscopy [42,43]. However, examinations of this type are known to be very invasive to the individual [44]. An alternative to these procedures takes the form of analyzing the voice signal—collected by a reading device with a microphone—and some of its acoustic parameters in order to infer its normality, or on the contrary, to detect a possible pathology in the voice. As an example, the “Italian Society of Phoniatrics and Speech Therapy”, or Società Italiana di Foniatria e Logopedia (SIFEL) [45], classifies the quality of an individual’s voice based on the analysis of characteristics associated with the signal, namely the fundamental frequency [46], the jitter and shimmer variations [47], and the harmonic-to-noise ratio (HNR) [48]. However, the manual classification of voice quality through the analysis of these parameters is very dependent on the algorithm performance that estimates these metrics and the clinical professional responsible for examining them [49,50], which makes the scalability of this procedure difficult, making it subjective, and consequently, reducing its accuracy. Thus, in view of the increased accuracy in detecting the presence of a disorder in a patient’s voice through the investigation of parameters associated with the sound signal, techniques based on the extraction of signal features and the classification of these features with machine learning methods have become popular on the topic in recent years, as demonstrated in the review works of Al-Hussain et al. [29] and Hegde et al. [30].

It is possible to computationally estimate many characteristics associated with the human vocal tract and the respective voice produced that can be used to infer the presence of dysphonia in an individual’s voice. For example, the characteristic known as pitch [51], which consists of a metric that estimates the opening and closing frequency of the vocal cords during speech production, was used by ElBouazzaoui et al. [52] to define a descriptor composed of up to 49 metrics extracted from this feature. Abnormalities in the vocal apparatus may present disturbances and instabilities associated with the sound signal in terms of frequency, defining the metric known as jitter; and amplitude, defining the metric known as shimmer, and these characteristics were used in the definition of pathology detection systems in the voice [53]. Metrics associated with estimating the proportion of noise that makes up the voice signal, such as the HNR, have also been successfully used in these systems. Teixeira et al. [54], for example, propose to represent the voice signal with four jitter characteristics, four shimmer characteristics, and the HNR measure for training an artificial neural network (ANN), which achieves an accuracy of up to 100% for classifying female voices. Still in this sense, Fernandes et al. [55] propose the use of a voice pathology detection system with low computational cost, whose operation is based on the autocorrelation metrics of time series, HNR, and on the noise-to-harmonic ratio (NHR). Features that aggregate time–frequency information of a signal, such as entropy, energy, and zero crossing rate (ZCR), were used to adjust a methodology based on paraconsistent discrimination engineering (PDE) to classify speech samples with different pathologies [56].

A category of features that have been used for a long time in voice pattern recognition, especially considering disorders, are those based on the wavelet concept [57,58]. Specifically, the discrete wavelet transform (DWT) is traditionally used for making features based, for example, on the signal energy in the frequency domain, as performed by Tsanas et al. [59] to detect the presence of Parkinson’s by speech analysis. Similarly, the DWT was used to compose a representation proposed by Fonseca et al. [60] that takes into account a version filtered by an inverse linear predictive filter (ILPF) of the voice signal to detect laryngeal infections from samples from a Brazilian database. Hammami et al. [61] also used parameters obtained by DWT with the application of the concept of empirical mode decomposition (EMD), together with high-order statistics (HOS), to detect voice pathologies in a two-step classification scheme. Saeedi and Almasganj [62] propose a representation version of the voice signal by measurements based on the energy of a wavelet transform whose parameters are optimally adapted by a genetic algorithm (GA), being evaluated positively in the classification of six voice disorders. In addition, frequency domain sub-band analysis using the stationary wavelet transform (SWT) performed by Gidaye et al. [32], proved to be effective in representing voice pathologies in several databases. Similarly, Shrivas et al. [31] constructed energy-based features and SWT statistical mappings to detect speech dysphonia. Furthermore, Kassim et al. [63] considered as characteristics of the signal the entropy of Tsallis [64] of dual-tree complex wavelet transform (DTCWT) [65].

Representations obtained from the cepstral domain or computed from features known as cepstral coefficients of a voice signal were used to detect patterns  [66], in particular the presence of pathologies [67], with MFCC [68] the most well-known technique in this category. The work of Godino-Llorente and Gómez-Vilda [69], for example, is one of the first advances that proposes the use of cepstral characteristics calculated from the MFCC and its first- and second-order derivatives, in the case of Δ and ΔΔ, to detect the presence of voice disorders. Arias-Londono et al. [70] used the cepstral coefficients of an MFCC to train a Gaussian mixture model (GMM), and used information from the modulation spectrum of the signal to train a support vector machine (SVM) and be able to form an ensemble of classification and detection of voice disorder. Cordeiro et al. [71] analyzed the speech signal’s MFCC together with their spectral line frequencies and used these features to train three classifiers: a GMM, an SVM, and a discriminant analysis machine. In [72]’s work, the Mel-scale spectrogram and the chroma features were used together with the signal’s MFCC to train a deep neural network (DNN), which enabled the development of a framework capable of detecting the presence of cordectomy in speech with an accuracy of 96.77%. In this sense, Lee [37] conducted a robust experimental analysis on HOS features together with cepstral features extracted from MFCC and LPCC to adjust a feedforward neural network (FNN) and a convolutional neural network (CNN). As an extension to the last work, Lee and Lee [73] added experiments considering data balancing techniques on the Saarbruecken voice database.

In summary, in Table 1, similarities and differences are presented about the main voice disorder detection works discussed in this section. Specifically presented are the analyzed work; the features used in the work to represent the voice signal; the method of dimensionality reduction or feature selection employed; the classifiers used to define the model; the considered voice database; the best accuracy percentage obtained by the model in some specific voice database selection, for example, with respect to gender or vowel; and the work publication year.

Given the above, it is clear that much has been carried out to overcome the problem of voice dysphonia detection through the analysis of handcrafted features and the use of machine learning techniques. However, this problem still remains open, since none of the existing methods presents 100% accuracy in all situations. In fact, some methods obtain such performance, but only in situations specific to the gender or to a section of the database, among others. Thus, developments on this topic are still needed. In addition, most studies that use cepstral features consider only one CC extraction technique, usually the MFCC, thus making analyses involving more than one cepstral feature in the representation of the voice signal scarce. Furthermore, it is not rare that the projection method used to convert the CCs used from their matrix form to their vector form is omitted in current works. In this work, we will analyze a set of eight cepstral feature extraction techniques and observe their operations along with an extensive set of noncepstral characteristics. Specifically, we will formalize the experimental analysis by considering various projection techniques over the cepstral domain and comparing each performance with respect to the problem of voice dysphonia detection. Thus, we highlight that the main differential of our work consists of the definition of a process that promotes the analysis of a set of CC extraction techniques in order to evaluate their ability to represent voice signals on the dysphonia detection problem. For this, we propose a new framework composed of stages of the multiprojection of CCs, fusion with noncepstral features, dimensionality reduction, data balancing, and model classification training to make possible the detection of dysphonia in speech signals.

## 3. Cepstral Features Extraction Fundamentals

**Figure 1:**
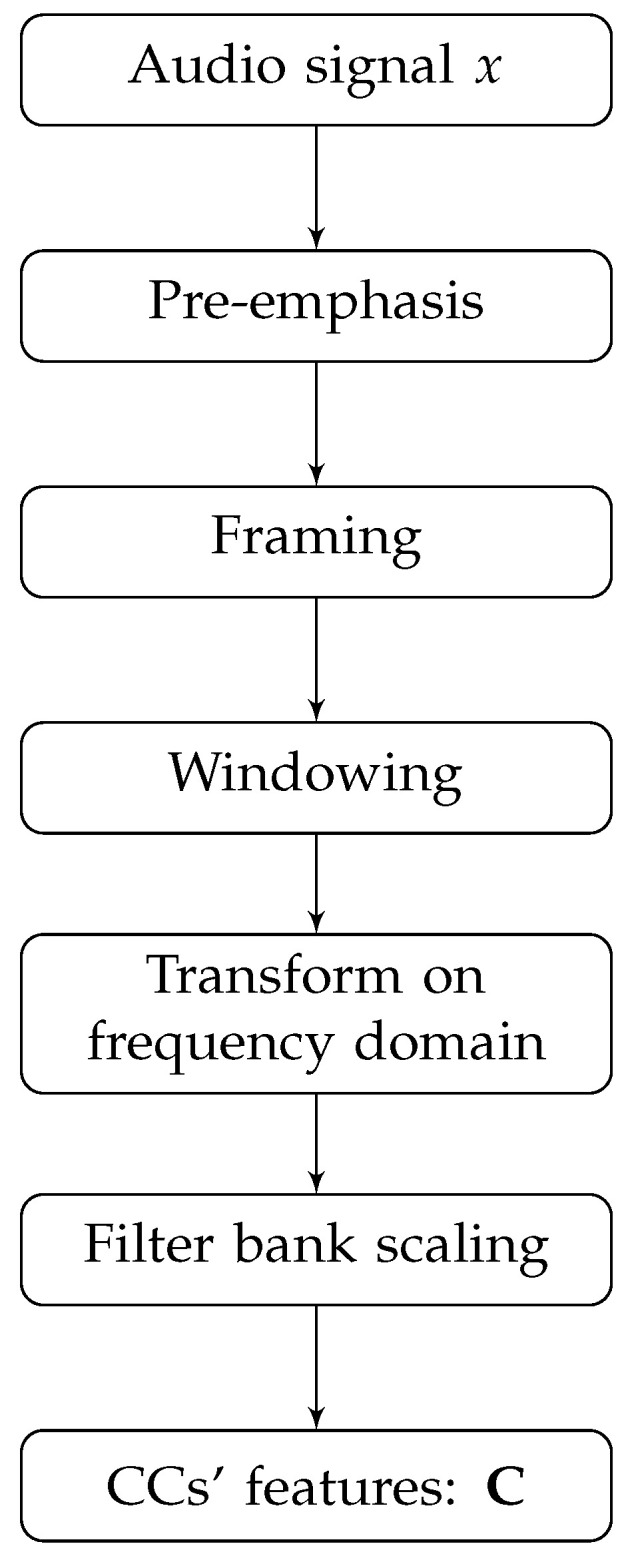
Generalization of cepstral feature extraction process based on CCs from an audio signal *x*.

To define a classification model based on the representation of the voice signal, it is necessary to use techniques that extract important characteristics of the analyzed signal for the considered problem. This usually involves a feature extraction step that highlights patterns from inherent features in the raw signal. In the area of audio processing and analysis, it is common to use features extracted directly from the temporal version of the sound signal, the most used being those based on energy [74], entropy [75], ZCR [76], and energy Teager operator (ETO) [77]. However, for problems that require a higher level of detail in the representation of the signal, as is the case of the problem of detecting dysphonia in the user’s speech, it may be more appropriate to use a category of features capable of representing harmonic characteristics and associated sidebands to the signal and its spectral domain. Examples of its techniques include those based on the use of the cepstral features of the signal, such as the MFCC and the LPCC, which are the representations analyzed in depth in this work. These techniques involve a pre-emphasis step to compensate for high-frequency suppression, followed by steps of splitting and filtering the signal to represent it in the frequency domain through some specialized transformation, such as the fast Fourier transform (FFT). Finally, CCs are calculated using a filter bank scale. Figure 1 presents a general outline of the CC calculation process in sound signals.

We can use the Φ technique to extract $n_{\mathrm{ceps}}$ CCs from each sound signal $x\in R^{n}$, considering $n_{\mathrm{frames}}$ frames of time. Mathematically, these CCs can be represented as a matrix $C_{x}^{\Phi }\in R^{n_{\mathrm{ceps}}\times n_{\mathrm{frames}}}$, according to Equation (1).

(1) $$C_{x}^{\Phi }=\left[\begin{array}{ccc} — & {\vec{c}}_{1} & —\\ — & {\vec{c}}_{2} & —\\ \; & \vdots & \\ — & {\vec{c}}_{n_{\mathrm{ceps}}} & — \end{array}\right]\in R^{n_{\mathrm{ceps}}\times n_{\mathrm{frames}}},$$

in which ${\vec{c}}_{i}\in R^{n_{\mathrm{ceps}}}$, for all *i*, are the CCs extracted from *x* using the technique Φ. More information about the mathematical foundation and implementation of these methods can be found in the works of Prabakaran and Shyamala [66], Alim and Rashid [78].

## 4. Methodology

**Figure 2:**
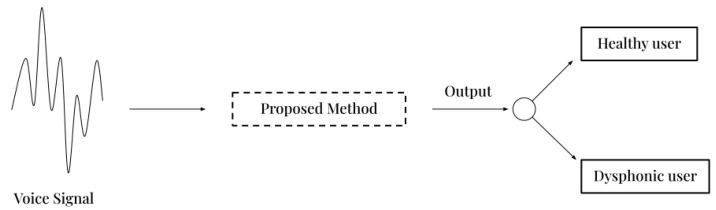
Scheme of the operation of the proposed material. Our method was developed to detect the presence of dysphonia in a user’s voice. Thus, user recognition routines are not part of our proposal.

Solutions for dysphonia detection can act according to different methodologies. However, two more common methodologies involve the definition of a user recognition system: the one that counts with all the steps of a BAS, and the one that defines only the voice disorder check step. In this work, we adopt the second. That is, the proposed material is only responsible for answering whether a voice signal belongs to a healthy individual or if it was obtained from an affected one. Thus, our operation must be restricted to the solution of a binary classification problem composed of vectors that can be added to two different classes: the group of users with a healthy voice; and the group of users with voice dysphonia. A representation of the acting of the proposed material is schematized in Figure 2.

In detail, the proposed methodology is defined by the four steps described below:

- **$M_{1}$—problem domain definition**: It is necessary to provide a voice signal recorded with a microphone device, in which we can suppose that there is a possibility of use from an affected individual. Thus, the problem domain is formed by voice signals that are usually defined in a time domain, that is, vectors in the space $R^{n}$.
- **$M_{2}$—proposed method**: As mentioned, in this work, the advances are contained in the dysphonia detection in a voice signal. Then, our contributions must compose or define specialized models to provide an answer to the VAS with respect to the type of voice signal that was provided to the system. For this, two new technologies are proposed to perform this task:
  − **$C_{1}$—multiprojection of cepstral features set**: This contribution consists of a mathematical formalization of representing a set of cepstral features that must be defined by matrices. Thus, this contribution is a generalization of representing a voice signal in a feature space, therefore configuring a handcrafted feature technique. Therefore, to circumvent the VAS performance loss problem caused by the possible presence of dysphonia in the signal, this contribution needs other operational steps, such as the use of a classifier.
  − **$C_{2}$—framework to increase the accuracy of dysphonia detection using machine learning**: Unlike $C_{1}$, this contribution corresponds to all dysphonia detection routines in the voice signal, as the use of a classifier is one of its steps. In this case, the framework is a proposal of steps that a dysphonia detection solution must perform. Furthermore, the proposed framework can be configured in different ways, as it is defined in a generalized way.
- **$M_{3}$—method output**: The developed tool must be able to point out if a given voice signal presents a sample recorded from a healthy person or from a dysphonic user. Thus, the method must work according to a binary classification routine, assigning one of the following values to the input signal: “healthy voice” or “dysphonic voice”.
- **$M_{4}$—validation**: To prove the effectiveness of the proposed material, analysis situations will be conducted considering the most used benchmark in the area, which is the Saarbruecken Voice Database. This database, which will be presented in more detail in the Experiments section, is formed by voice samples obtained from healthy individuals and from individuals affected by diseases that act directly on the human vocal tract. Specifically, for this work, we will consider voice samples from healthy individuals and voice samples from individuals classified as affected by “dysphonia”. In all test situations, what must determine whether a technique succeeds in the classification task are performance measures that are associated with the accuracy of the model. Furthermore, as the two contributions of this work allow for different configurations, several specific instances of the proposed material will be considered in all test scenarios. In detail, bearing in mind that the proposed model is a generalization, and consequently allows several specific instances, comparisons are conducted between many proposed instances.

## 5. Proposed Multicepstral Framework Based on Multiprojection Strategies for Voice Dysphonia Detection

In this section, we describe the elements that constitute the method developed to identify dysphonia in a voice signal. We thoroughly explain the operation of all the employed techniques through algorithms and flowcharts that aim to facilitate the understanding and reproduction of the developed material. In particular, we highlight the following innovations obtained with this work:

- A new framework for extracting and classifying the features of voice signals, with the aim of discriminating the samples into two distinct groups: the first group consists of speech samples from healthy individuals, while the second group contains speech samples from individuals suffering from of dysphonia;
- An experimental analysis of several configurations of the proposed generalization is conducted in this work. Furthermore, an important contribution is a computational evaluation of the performance of the representation of several features based on CCs, projected by different types of mappings, to solve the voice dysphonia detection problem, using three different classifiers.

### 5.1. Cepstral Feature Multiprojection

Mapping patterns given in their matrix form using a set of various functions have already been explored in other classes of pattern recognition problems. For example, in the case of spoofing detection problem in fingerprints, Contreras et al. [79] proposed a vector representation of statistical measures of the pattern descriptor dense scale-invariant feature transform (Dense-SIFT) [80] using five different mappings, even though it was originally given in matrix form. Furthermore, in a later work, Contreras et al. [81] generalized this concept to any matrix-based texture descriptor, using a set of mapping functions. In this work, we generalize this concept for the case of patterns described by CCs, which are also defined in a matrix form. It is also worth mentioning that this type of strategy is already used frequently in the area of sound processing. However, to the best of our knowledge, no work has formalized the concept of multiprojection and experimented with the problem of detecting voice dysphonia considering cepstral features. Thus, to enable such experimentation, we define the set M of mapping functions given in Equation (2):

(2) $$M=\left\{m_{1},m_{2},...,m_{n_{M}}\right\},$$

in which $m_{i}:R^{n_{\mathrm{ceps}}\times n_{\mathrm{frames}}}\to R^{n_{\mathrm{ceps}}},\forall i\in \left\{1,2,...,n_{M}\right\}$ is a function that projects the $n_{\mathrm{ceps}}$ CCs, originally belonging to the $R^{n_{\mathrm{frames}}}$ space, to the R space.

**Figure 3:**
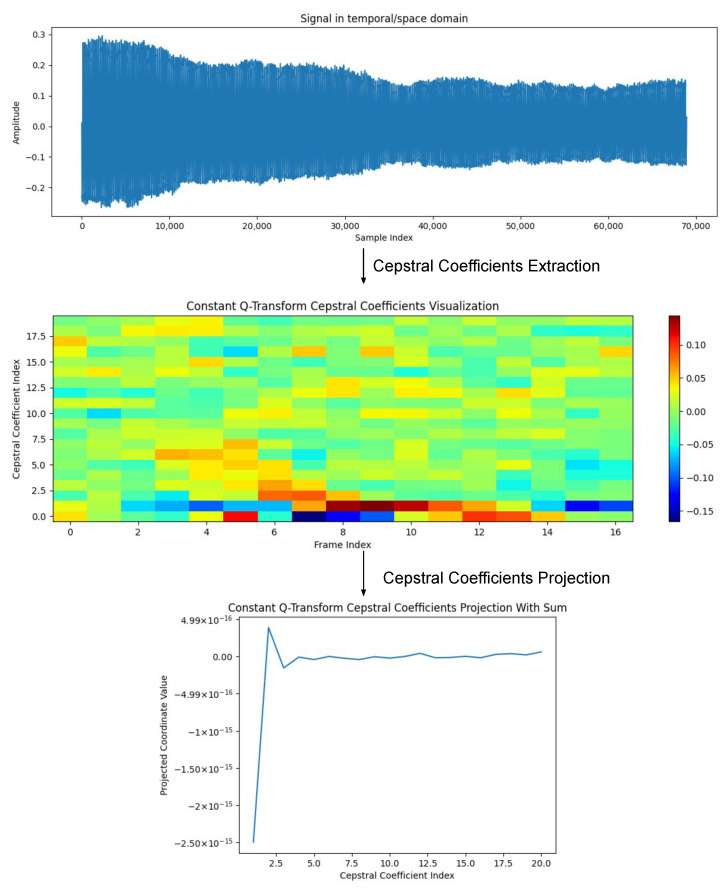
Tutorial scheme for the extraction of CCs from a voice signal considering the CQCC technique followed by the projection of these features by the sum mapping function. We can see, in this case, that the feature, originally belonging to the $R^{20\times 80}$ space, was projected to the $R^{20}$ space.

In Figure 3, we present an example of the projection process of a CC extracted from a voice signal *x*. In this case, the mapping function is the sum of the columns of the cepstral feature extracted by the constant Q cepstral coefficient (CQCC) [82] technique (Φ), which is originally given in the form of a matrix $C_{x}^{\Phi }\in R^{20\times 80}$, as in Equation (1).

So, if we have a matrix $C_{x}^{\Phi }$ of CC features generated by a technique Φ from the signal *x*, as shown in Equation (1), and their first- and second-order differentials represented, respectively, by $\Delta_{x}^{\Phi }$ and $\Delta \Delta_{x}^{\Phi }$. The definition of the cepstral features of *x* through the proposed multiprojection strategy, and with respect to the set M and method Φ, is expressed by the vector ${\vec{v}}_{x}^{M,\Phi }$, defined in Equation (3):

(3) $$\begin{array}{cc} {\vec{v}}_{x}^{M,\Phi }:= & A_{1}\left(m_{1}\left(C_{x}^{\Phi }\right),m_{1}\left(\Delta_{x}^{\Phi }\right),m_{1}\left(\Delta \Delta_{x}^{\Phi }\right),m_{2}\left(C_{x}^{\Phi }\right),m_{2}\left(\Delta_{x}^{\Phi }\right),m_{2}\left(\Delta \Delta_{x}^{\Phi }\right),...,\right.\\ & \left.\;m_{n_{M}}\left(C_{x}^{\Phi }\right),m_{n_{M}}\left(\Delta_{x}^{\Phi }\right),m_{n_{M}}\left(\Delta \Delta_{x}^{\Phi }\right)\right), \end{array}$$

in which $A_{1}$ is an information fusion strategy, such that ${\vec{v}}_{x}^{M,\Phi }\in R^{n_{\mathrm{proj}}}$. For the case of $A_{1}$ being a concatenation, which is the case evaluated in our experiments, the value of $n_{\mathrm{proj}}$ is equal to $3\cdot n_{\mathrm{ceps}}\cdot n_{M}$.

It is important to point out that using the proposed technique, it is possible to reduce the number of coordinates of the cepstral feature. Originally, this feature has $n_{\mathrm{ceps}}\cdot n_{\mathrm{frames}}$ coordinates; however, with the proposed technique, we can reduce it to $n_{\mathrm{ceps}}\cdot n_{M}$ coordinates. This reduction is generally efficient, since it is common for the number of frames considered in the representation of CCs to be very large, and therefore, much greater than $n_{M}$, which is the number of mapping functions that we will consider. In our method, this saving tends to be even greater, as we consider not only the static characteristics of the CCs, but also the dynamic characteristics, such as Δ and ΔΔ.

### 5.2. Extraction of Cepstral Features Set

CC extraction techniques are fundamental for the analysis and processing of speech signals. Several techniques can be used to extract CCs, and the choice of the most appropriate technique depends on the purpose of the application and the characteristics of the speech signal in question. Among the most common techniques, the MFCC and LPCC stand out. The MFCC is a widely used technique, which uses the Mel scale to map the frequencies of the speech signal, and employs the discrete Fourier transform (DFT) to extract the CCs. The LPCC, on the other hand, uses a linear predictive model to estimate the spectral coefficients, which are then transformed into CCs. Both techniques have advantages and disadvantages, and the choice between them depends on the characteristics of the speech signal and the needs of the application. The MFCC, for example, is considered more robust in noisy environments and has been widely used in speech recognition applications, while the LPCC is more sensitive to changes in the frequency spectrum, and has been used in applications for detecting changes in the signal. In summary, CC extraction techniques are essential for the analysis and processing of speech signals, and each technique is more suitable according to the characteristics of the speech signal and the needs of the application in question. Therefore, each technique has the capacity to more accurately represent a certain characteristic inherent to the voice signal, which can be better used for the representation and detection of a pattern that is determinant in each analyzed situation, since different CC extraction techniques can capture different aspects of the speech signal. The aggregation of CCs obtained through multiple techniques allows the speech recognition model to be more robust and generalizable since the relevant information is captured in a more complete and complementary way. In this context, in this work, we propose a voice signal representation step that consists of extracting and aggregating the cepstral features obtained from a set of $n_{P}$ CC extraction techniques, denoted by P, as shown in Equation (4).

(4) $$P=\left\{\Phi_{1},\Phi_{2},...,\Phi_{n_{P}}\right\},$$

in which $\Phi_{i}:R^{n}\to R^{n_{\mathrm{ceps}}\times {n_{\mathrm{frames}}}_{,i}}$ is a technique that extracts $n_{\mathrm{ceps}}$ CCs from the signal $x\in R^{n}$ considering ${n_{\mathrm{frames}}}_{,i}$ time frames.

Specifically, considering M as in Equation (1) and P as in Equation (4), we can generalize the procedure of the proposed framework for extracting multiprojected cepstral features from a voice signal $x\in R^{n}$ by the following steps:

1. Calculate the CCs of *x* using the techniques of P. That is, $C_{x}^{\Phi_{i}}:=\Phi_{i}\left(x\right),\forall \Phi_{i}\in P$;
2. Calculate the first- and second-order differentials of each CC $C_{x}^{\Phi_{i}}$ defined in the previous step, these being represented, respectively, by $\Delta_{x}^{\Phi_{i}}$ and $\Delta \Delta_{x}^{\Phi_{i}}$;
3. Carry out the multiprojection of the calculated cepstral features using Equation (3), and consequently, defining $n_{P}$ vectors ${\vec{v}}_{x}^{M,\Phi_{i}}$;
4. Aggregate the vectors ${\vec{v}}_{x}^{M,\Phi_{i}}$ using the information fusion strategy $A_{2}$ according to Equation (5):
  (5) $${\vec{v}}_{x,\mathrm{cepstral}}:=A_{2}\left({\vec{v}}_{x}^{M,\Phi_{1}},{\vec{v}}_{x}^{M,\Phi_{2}},...,{\vec{v}}_{x}^{M,\Phi_{n_{P}}}\right).$$

Even though the proposed generalization allows us to use any aggregation strategy for $A_{2}$, we will keep our experiments considering vector concatenation. Thus, ${\vec{v}}_{x,\mathrm{cepstral}}\in R^{n_{P}\cdot 3\cdot n_{\mathrm{ceps}}\cdot n_{M}}$.

### 5.3. Voice Signal Vector Representation

As seen in Section 2, there are noncepstral measures that are extremely useful in speech pattern recognition systems, especially in the case of voice pathology detection. This is due to the fact that noncepstral measures provide additional information about speech characteristics that may not be captured by cepstral measures. For example, jitter and shimmer measures are noncepstral measures that provide information about speech amplitude and frequency variation, respectively. These measures are important because they can be indicators of speech disorders, such as dysphonia. Other noncepstral measures, such as mean formant measures, can be used to characterize the pronunciation of different phonemes and help distinguish between different words. In addition, intensity and duration measurements can provide information about speech emphasis and rhythm, respectively, which can also be useful in detecting voice pathologies. By combining these noncepstral measures with cepstral measures, it is possible to obtain a more complete view of the features of the voice signal, and consequently improve the accuracy and reliability of the dysphonia detection process. Therefore, we propose that the voice signal is also represented by metrics obtained through noncepstral features. For this, let us consider the set N formed by $n_{N}$ metrics, not necessarily cepstral, that are able to represent some specific characteristic of a voice signal:

(6) $$N:=\left\{\mu_{1},\mu_{2},...,\mu_{n_{N}}\right\},$$

in which $\mu_{i}:R^{n}\to R^{n_{i}},\forall i\in \left\{1,2,..,n_{N}\right\}$.

Let us also define the feature vector ${\vec{v}}_{x,\mathrm{non}-\mathrm{cepstral}}$ of a speech signal $x\in R^{n}$ built from the fusion of information from the metrics of N applied to *x*:

(7) $${\vec{v}}_{x,\mathrm{non}-\mathrm{cepstral}}:=A_{3}\left(\mu_{1}\left(x\right),\mu_{2}\left(x\right),...,\mu_{n_{N}}\left(x\right)\right),$$

in which $A_{3}$ is a general information fusion strategy, but for experimentation level, let us consider it to be the concatenation of vectors. Finally, to represent the voice signal with its cepstral and noncepstral features, we propose the use of the vector ${\vec{v}}_{x}$ as defined in Equation (8) below:

(8) $${\vec{v}}_{x}:=A_{4}\left({\vec{v}}_{x,\mathrm{cepstral}},{\vec{v}}_{x,\mathrm{non}-\mathrm{cepstral}}\right),$$

in which $A_{4}$ is another information fusion strategy, but which will also be considered a vector concatenation.

### 5.4. Dysphonia Detection Model Definition

If we consider that $A_{1}$, $A_{2}$, $A_{3}$, and $A_{4}$ are fusion strategies that consist of the concatenation of vectors, then ${\vec{v}}_{x}$ has $n_{P}\cdot 3\cdot n_{\mathrm{ceps}}\cdot n_{M}$ coordinates from cepstral features and $\sum_{i=1}^{n_{N}}n_{i}$ coordinates from noncepstral features. Thus, in many configurations of the proposed framework, it is expected that the vector ${\vec{v}}_{x}$ has a high dimension, which can result in the curse of dimensionality for this representation. Consequently, the accumulation of noise and redundant information can affect the representation capability of ${\vec{v}}_{x}$. To work around this situation, it is suggested the addition of a step in which some technique of data dimensionality reduction is employed, represented by the transformation REDUCTION(·), which maps data from a space $R^{n_{\mathrm{high}}}$ to a space $R^{n_{\mathrm{low}}}$, where $n_{\mathrm{low}}<<n_{\mathrm{high}}$.

Finally, it is necessary to establish a dysphonia detection model using a classifier. In this case, it is necessary to use a base $B_{\mathrm{Train}}$ of features extracted from voice samples related to training, as presented in Equation (9), to fit a classification algorithm. Furthermore, it is common to apply a normalization strategy on the feature vectors along with the classification algorithm. For this, we consider in our framework that the normalization strategy NORM(·) is used.

(9) $$B_{\mathrm{Train}}:=\left\{{\vec{v}}_{x_{1}},{\vec{v}}_{x_{2}},...,{\vec{v}}_{x_{n_{T}rain}}\right\},$$

where $x_{i}$, $i\in \{1,2,..,n_{\mathrm{Train}}\}$, is a sample in the considered training voice database and $n_{\mathrm{Train}}$ is the total number of samples.

Furthermore, we must also take into account that in many situations involving voice pathology detection, most of the available databases scarcely present us with a balanced distribution of examples belonging to each class. As an example, the Saarbruecken voice database itself presents us with a number of distinct examples of voices from healthy individuals and individuals affected by dysphonia, as detailed in Section 7. Therefore, we also propose that a data balancing routine be adopted on the feature vectors database $B_{\mathrm{Train}}$ prior to dimensionality reduction and normalization processes. Therefore, these processes will be applied directly to a balanced database, that is, a database containing the same number of feature vectors of healthy and pathological individuals.

In summary, we describe in Algorithm 1 the operation of the voice dysphonia detection model in the proposed framework using a base of feature vectors extracted from speech samples with and without the presence of dysphonia.
**Algorithm 1** Classifier training stage and definition of the dysphonia detection model in the proposed framework.    $B_{\mathrm{Train}}$  Training database with $n_{\mathrm{Train}}$ feature vectors. **Input:**  REDUCTION(·)   Dimensionality reduction strategy.    NORM(·)    Normalization function.1:Application of a data balancing strategy on $B_{\mathrm{Train}}$.2:**for**$i\in \left\{1,2,3,..,n_{\mathrm{Train}}\right\}$**do**3:    ${\hat{v}}_{x_{i}}:=\mathrm{NORM}\left(\mathrm{REDUCTION}\left({\vec{v}}_{x_{i}}\right)\right)$▹ Reduce the dimension of feature vectors and normalize them.4:**end for**5:${\hat{B}}_{\mathrm{Train}}:=\left\{{\hat{v}}_{x_{1}},{\hat{v}}_{x_{2}},...,{\hat{v}}_{x_{n_{\mathrm{Train}}}}\right\}$▹ Define a training database normalized with feature vectors with smaller dimension.6:Train a classifier with ${\hat{B}}_{\mathrm{Train}}$. **Output:** The proposed dysphonia detection model.

### 5.5. Proposed Algorithm

The proposed framework involves the sequential and combined use of all the steps described in the previous subsections. In detail, the execution of the following processes is established:

1. Establish the necessary parameters for the execution of the framework. For example, the mapping functions that will compose the set M, the CC extraction techniques, and the used classifier, among other configurations.
2. Extract the CCs and their first- and second-order differentials from each available voice sample using all the techniques in the P set.
3. Conduct the proposed multiprojection process to build the cepstral feature vector $v_{x,\mathrm{cepstral}}$ for each speech signal *x*.
4. Construct the vector of non-cepstral features for each voice sample.
5. Aggregate cepstral and non-cepstral information for each voice signal, building a feature vector database.
6. Conduct balancing of the training feature vectors such that the number of vectors associated with pathological individuals should be the same number of vectors associated with healthy individuals.
7. Reduce the dimensions of the made-up feature vectors.
8. Normalize the reduced-dimensional feature vectors defined in the previous step.
9. Train a classifier based on normalized and reduced-dimensional feature vectors.

**Figure 4:**
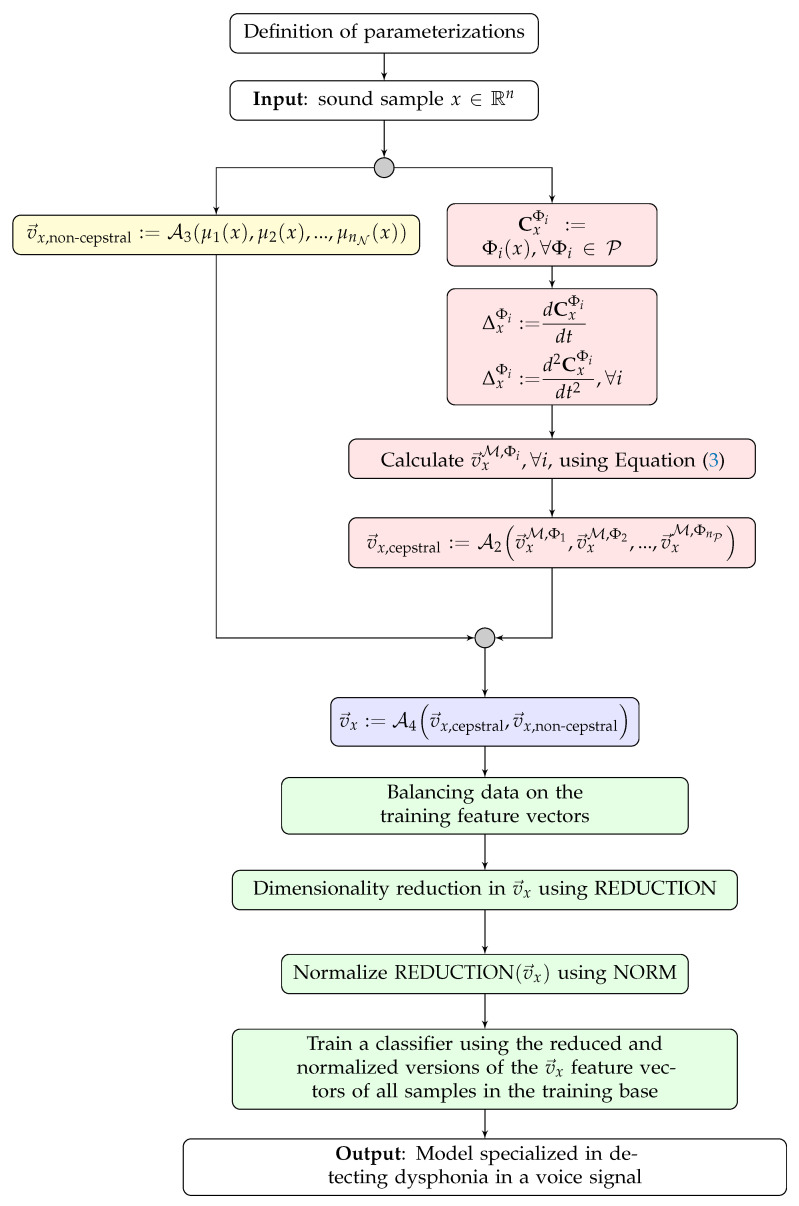
Flowchart of the proposed framework. The colors represent the steps of the method. Specifically, the white color defines setup and procedure input and output steps; yellow represents the step of extracting noncepstral features from the voice signal; red represents the extraction of multiprojected cepstral features from the signal; blue represents the aggregation of all calculated features; and the green color represents the definition step of the dysphonia detection model in a speech signal.

Finally, in Figure 4, a flowchart is presented that gathers the processes described in this section and represents the execution of the proposed method steps.

Due to the general nature of the proposed contributions, the parameters of the method detailed here can be adjusted in order to represent some techniques from the specialized literature. For example, the work of Dankovičová et al. [34] is a special case of our modeling, which can be obtained according to the following specific configuration:

- The set N of noncepstral features is represented by the following measures: energy, low-short time energy ratio, ZCR, Teager–Kaiser energy operator, entropy of energy, Hurst’s coefficient, $F_{0}$, formants, jitter, shimmer, spectral centroid, spectral roll-off, spectral flux, spectral flatness, spectral entropy, spectral spread, linear prediction coefficients, HNR, power spectral density, and phonatory frequency range.
- The cepstral features are defined solely by the static CCs calculated by the MFCC technique. In other words, P={MFCC}.
- The CCs are projected using mean. Thus, M={MEAN}.
- All the features are fused using concatenation. Thus, $A_{1}=A_{2}=A_{3}=A_{4}$ = CONCATENATION.
- No data balancing strategy is cited, so this procedure should be disregarded.
- Two dimensionality reduction strategies are considered: one based on feature selection by mutual information, and another based on the PCA.
- As no data normalization routine is mentioned, the NORM function must be the identity function. That is, NORM(y)=y, for all *y*.
- Three classifiers are considered: SVM, RF, and KNN.

Thus, we can see that the proposed method has a great capacity for generalization and adaptation, being able to define a wide variety of techniques from the adjustment of its parameters. Therefore, in the next section, we will define some practical instances of the proposed material to make it possible to evaluate the dysphonia detection problem.

## 6. Parameters for the Proposed Method and Practical Instances

It is worth noting that all proposed contributions—both the operation of multiprojection of cepstral features and the stages of the voice dysphonia detection framework—were presented in the form of generalizations. That is, the proposed representation is dependent on a set of mapping functions M, a fusion strategy $A_{1}$, and a CC extraction technique. Similarly, the framework is dependent on sets of CC extraction techniques, feature fusion strategies, and data balancing methods, among other parameters. Therefore, it is necessary to establish a specific configuration for both contributions in order to obtain a usable instance. To carry out the experiments, the objective was to define the simplest possible parameterization for each case, in order to evaluate the effectiveness of the proposed strategies with elementary and accessible methodological additions. Therefore, we will establish below the techniques that, in fact, define the functioning of the contribution stages:

- **Fusion strategies**: As already mentioned in the description of the steps of the proposed method, all information fusion strategies will be defined as the concatenation of vectors. Therefore, $A_{1}=A_{2}=A_{3}=A_{4}=\mathrm{CONCATENATION}$.
- **Mapping functions**: For our experiments, we will consider four matrix projection strategies, as defined in Equation (3): the projection of columns by PCA ($m_{\mathrm{PCA}}$); the sum of columns ($m_{\mathrm{SUM}}$); the standard deviation of columns ($m_{\mathrm{STD}}$); and the skewness of the columns ($m_{\mathrm{SKEW}}$). We will also consider some combinations of these mappings to employ the proposed multiprojection concept. Specifically, let us consider the sets of mappings defined in Table 2.
- **Noncepstral features**: As noncepstral measures are associated with the voice signal that must compose the set N, the following will be considered [23,83,84,85,86,87,88,89]:
  − ”Statistical moments of the fundamental frequency”: In this work, we will use only the mean and standard deviation of $F_{0}$.
  − “HNR”: As mentioned in Section 2, this is an acoustic measurement that compares the energy ratio of harmonic and noise components in a speech signal. Generally, the HNR is used as an indicator of voice quality, with higher values indicating a clearer, less noisy voice.
  − “Local Jitter”: This is the time difference between the actual duration of each audio frame and the expected average duration of each frame.
  − “Local Absolute Jitter”: This is a measure of time variation similar to Local Jitter that is calculated from the absolute differences between the time intervals between consecutive voice frames and the average of these intervals.
  − “Relative Average Perturbation (RAP) Jitter”: This is a measure that describes the relative variation between the durations of the time intervals between consecutive speech signal cycles. In other words, Rap Jitter is calculated as the ratio between the standard deviation of the time intervals and the average of the time intervals.
  − “Local dB Shimmer”: This is used to assess variability in sound wave amplitude during speech. The Local dB Shimmer measure is calculated as the local frame-by-frame variation in the amplitude of the sound wave, expressed in decibels (dB). Specifically, it is the difference between the maximum and minimum value of the sound wave amplitude in a specific voice frame, expressed in dB.
  − “Amplitude Perturbation Quotient 3 (APQ3) Shimmer”: Calculates the change in the amplitude of the sound wave during speech using the difference between the amplitude values at three equally spaced points in a speech cycle. The APQ3 Shimmer is calculated as the mean of the absolute differences between the amplitude values at three consecutive points divided by the mean amplitude value at those three points.
  − “APQ5 Shimmer”: This is similar to the APQ3 Shimmer, but uses five equally spaced points on a voice cycle to calculate the change in the amplitude of the sound wave.
  − “APQ11 Shimmer”: A measure similar to APQ3 Shimmer and APQ5 Shimmer, but considering 11 equidistant points.
  − “DDA Shimmer”: This is calculated as the average of the absolute differences between the sound wave amplitude values in a voice cycle, divided by the number of samples in the cycle. The difference between the amplitude values is calculated as the absolute difference between the maximum and minimum amplitude values in the voice cycle.
  − “Jitter PCA projection”: A one-dimensional PCA projection of all Jitter-based features that were used.
  − “Shimmer PCA projection”: A one-dimensional PCA projection of all Shimmer-based features that were used.
  − “Fitch Virtual Tract Length (FVTL)”: This involves analysis of the acoustic spectrum of the sound produced during speech and the comparison with mathematical models that relate the acoustic properties with the length of the vocal tract.
  − “Mean and median of the four formant frequencies”: $F_{1}$, $F_{2}$, $F_{3}$, and $F_{4}$ are the four main formant frequencies that are measured and analyzed in speech processing. $F_{1}$ is the lowest frequency of the first formant, which is determined by the position of the jaw and tongue. $F_{2}$ is the frequency of the second formant, which is mainly influenced by the position of the tongue in the mouth. $F_{3}$ and $F_{4}$ are the frequencies of the third and fourth formants, respectively, and are mainly influenced by the opening of the lips and the position of the soft palate.
  − “Formant Dispersion”: One-third the value of the difference between the medians of the fourth and first formants.
  − “Arithmetic mean of formant frequencies”: Mean between the medians of the first four formant frequencies.
  − “Formant Position”: Standardized mean of the medians of the first four formant frequencies.
  − “Spacing between formant frequencies (ΔF)”: Estimation of minimum spacing between formant frequencies using linear regression.
  − “VTL of ΔF”: The spacing between any two consecutive formants in the frequency spectrum, which can be estimated by the speed of propagation of sound in air divided by twice the spacing between the formant frequencies.
  − “Mean formant frequency (MFF)”: Fourth root of the product between the medians of the first four formant frequencies.
- **Cepstral features**: For CC extraction techniques, the following methods will be considered: CQCC, MFCC, inverse MFCC (iMFCC), linear-frequency cepstral coefficients (LFCC) [90], gammatone-frequency cepstral coefficients (GFCCs) [91], bark-frequency cepstral coefficients (BFCCs) [92], LPCC [93], and normalized gammachirp cepstral coefficients (NGCC) [94], all of which are defined with $n_{\mathrm{ceps}}=20$ static coefficients, 20 first-order dynamics (Δ) and 20 s-order dynamics (ΔΔ), being normalized by mean and variance, which configures the strategy of cepstral mean and variance normalization (CMVN). These techniques are considered separately and in combination according to the sets defined in Table 3. Due to text space limitations, it was not possible to consider all existing combinations of the eight CC extraction techniques. However, the most important combinations were analyzed to evaluate the performance of the developed material. We consider versions of $P_{1}$ to $P_{8}$, in which each representation of P has only one element. These versions allowed for an evaluation of how much the framework is enhancing the capacity of the techniques to detect dysphonia in voice signals. In addition, the other versions of CC extraction techniques should serve to confirm the ability of the proposed material to represent different features in the sound sample, which should contribute to improving its ability to detect dysphonia in these samples.
- **Balancing data**: As a data balancing technique, we chose to adopt an oversampling strategy on the minority set of samples in order to balance the number of representatives of each class. Specifically, we use one of the simplest techniques for this purpose: the synthetic minority oversampling technique (SMOTE).
- **Dimensionality reduction**: The singular value decomposition [95] technique will be used as a REDUCTION(·) function, as it is considered one of the simplest and most representative techniques of the dimensionality reduction methods. It is worth mentioning that this technique, which is very similar to the well-known PCA, is based on the representation of the data through a new basis constituted by the eigenvectors associated with the eigenvalues of larger modules, which represent the variances of the original data in the directions of these vectors. This reduced-dimensional representation tends to decrease the covariance, and consequently, the redundancy [96,97] between the considered data, which can be beneficial in the ${\vec{v}}_{x}$ representation, since the feature fusion strategies we are using are based on the concatenation of vectors. For each considered version, four dimensions will be evaluated to compose the reduced feature space. This includes a version reduced to 10% coordinates, another reduced to half coordinates, another reduced to 75% coordinates, and a version without reduction. These dimensions represent different levels of feature space dimensionality reduction, allowing us to evaluate how the reduction affects the technique’s ability to detect dysphonia in voice signals.
- **Normalization**: Four different strategies were used to normalize the feature vectors, considered the most common in the addressed problem. For each considered version, four different normalizations were evaluated: the Min–Max normalization ($NORM_{\mathrm{MM}}$), standard normalization ($NORM_{\mathrm{STD}}$), robust normalization ($NORM_{\mathrm{ROB}}$) and nonuse of normalization ($NORM_{\mathrm{UnS}}$). Mathematically, the training basis of the feature vectors in the reduced space $R^{m}$ is represented by ${\hat{B}}_{\mathrm{Train}}:=\left\{{\hat{v}}_{x_{1}},{\hat{v}}_{x_{2}},...,{\hat{v}}_{x_{n_{\mathrm{Train}}}}\right\}$, where each vector ${\hat{v}}_{x_{j}}=\left({\hat{v}}_{x_{j},1},{\hat{v}}_{x_{j},2},...,{\hat{v}}_{x_{j},m}\right)$. Specifically, the four considered normalization functions are:
  1. Min–Max scale (MM):
    $$NORM_{\mathrm{MM}}\left(y\right):=\left(\frac{y_{1}-\min_{j}\left\{{\hat{v}}_{x_{j},1}\right\}}{\max_{j}\left\{{\hat{v}}_{x_{j},1}\right\}-\min_{j}\left\{{\hat{v}}_{x_{j},1}\right\}},\cdots ,\frac{y_{m}-\min_{j}\left\{{\hat{v}}_{x_{j},m}\right\}}{\max_{j}\left\{{\hat{v}}_{x_{j},m}\right\}-\min_{j}\left\{{\hat{v}}_{x_{j},m}\right\}}\right);$$
  2. Standard scale:
    $$NORM_{\mathrm{STD}}\left(y\right):=\left(\frac{y_{1}-\mu_{1}}{\sigma_{1}},\cdots ,\frac{y_{m}-\mu_{m}}{\sigma_{m}}\right),$$
    where $\mu_{i}=\sum_{j=1}^{n_{\mathrm{Train}}}\frac{{\hat{v}}_{x_{j},i}}{n_{\mathrm{Train}}}$ and $\sigma_{i}=\frac{1}{\sqrt{n_{\mathrm{Train}}}}\sqrt{\sum_{k=1}^{n_{\mathrm{Train}}}{\left({\hat{v}}_{x_{k},i}-\mu_{i}\right)}^{2}};$
  3. Robust scale ($NORM_{\mathrm{ROB}}$): Robust normalization is an adaptation of standard normalization that handles extreme values in the data more efficiently. This approach replaces the mean of the values ($\mu_{i}$) with the median, which is less sensitive to these extreme values; and the standard deviation ($\sigma_{i}$) with the interquartile range, which is the difference between the first and the third quartile of the distribution. This makes normalization more resistant to outliers that can degrade model performance.
  4. No scale: this is the strategy represented by the identity function. That is, $NORM_{\mathrm{UnS}}\left(y\right)=y$.
- **Classifiers**: We will evaluate the three most used classifiers in the addressed problem. Specifically, let us consider a Gaussian kernel SVM [98], also known as a radial basis function (RBF) kernel, an RF [99], and a linear logistic regression (LR) [100]. All of these classifiers are flexible in the sense of allowing an unbalanced data-resistant configuration. We will consider this configuration even in situations where we do not use SMOTE on the feature vector database.

## 7. Results and Experiments

In this section, we will carry out the necessary tests to evaluate the performance of the advances presented in Section 5. For this, a benchmark is defined, described in detail in Section 7.1, which is widely recognized in the field in question. In the specific case of this study, it is necessary to perform an internal analysis of the proposed content to evaluate the effectiveness of the proposed method in relation to the various configurations mentioned in Section 6. To make comparisons between the performances of the various instances of the proposed material, it is necessary to establish some performance metrics that represent the amounts of errors and successes of the method in relation to the samples of voice signals from the test bases. In this study, the following metrics [101] were used:

- Error in Healthy (EH): represents the rate of healthy voice signals classified as pathological.
- Error in Pathological (EP): represents the percentage of pathological voice signals classified as healthy.
- Equal Error Rate (EER): the average between the false-positive and false-negative rates of the method. Mathematically,
  $$EER=\frac{EH+EP}{2}.$$
- Accuracy (ACC): the rate of correctly classified voice signals.
- F1-score: the harmonic mean between the sensitivity and accuracy of the model.
- K-fold Cross-Validation Score (KFCV): Average accuracy score of a 5-fold cross-validation over the training dataset.

All the implementations presented in this work were coded in Python (https://www.python.org/ (accessed on 22 May 2023)) programming language, more specifically with the use of scikit-learn [102], Spafe [103], and Praat-Parsemouth [104], on a personal microcomputer equipped with 8 GB of RAM and an Intel (R) Core (TM) i5-4460 of 3.20 GHz frequency.

### 7.1. Benchmark

To compose the benchmark of the experiments carried out in this work, we used the well-known Saarbruecken Voice Database [40], one of the most used and most challenging voice sample databases in voice pathology detection problem. In detail, this database has voice signals collected from more than 2000 individuals, which are sampled at a frequency of 50kHz with 16-bit resolution and with 1 to 2 s. The database was created and is maintained by the Institute of Phonetics of the University of Saarland (https://stimmdatenbank.coli.uni-saarland.de/help_en.php4 (accessed on 22 May 2023)). For each individual, their speech is recorded by reproducing the vowels \a\, \i\, and \u\ at normal, high, and low pitch. Moreover, a varying low–high–low pitch is considered. Also available in this database is a recording of a sentence in German and the individual’s electroglottograph signals, but these will not be considered in this work. Specifically, for our experiments, we exported a section of this database referring only to the signals associated with the pathology classified as “dysphonia”, which is the voice disorder addressed in this study. Requiring only individuals over 18 years, the database provided us with the exact following distribution of voice signals:

- 384 recordings of healthy male subjects;
- 1524 recordings of healthy female subjects;
- 324 recordings of dysphonic male subjects;
- 492 recordings of dysphonic female subjects.

We can observe that the considered clipping has a much larger set of voice signals from healthy female individuals than from any other condition or gender, which sets up a situation of information imbalance, making challenging the problem of defining a robust model to voice dysphonia detection. Finally, following an existing trend in the works reviewed in Section 2, we randomly split the obtained dataset into 75% for model training and fit signals and 25% for method testing and evaluation. It is worth mentioning that this division is used to analyze the method’s performance according to the ACC, F1 and EER techniques, since to investigate the KFCV metric, the entire database clipping is analyzed, and different splittings between the training and test sets are dynamically established.

### 7.2. Performance Analysis

As described in Section 6, the proposed material allows for multiple configurations, with around 26,000 variations having been evaluated in this work, which resulted in more than 1 million analysis performance observations on the selected voice database. The complete record of these evaluations can be found in the complementary materials to this study that are available in the respective Zenodo repository [105]. In this section, we will present a summary of all these results, highlighting the best performances in each case and the phenomena that prove the effectiveness in the use of the proposed material. We started this discussion with the results arranged in Table 4, in which we present the best values, according to each considered metric, presented by one of the configurations of our method. For this, we present the results according to the vowel uttered by the evaluated individual and the respective intonation. We also sectioned the results according to the gender of the individuals, considering a partition composed only of male voices, a partition composed only of female voices, and a partition that considers both groups of voices. In each instance, the following are presented, respectively: the best value of the analyzed metric among all the configurations considered for the proposed method for the partition in question; the type of feature that represents the voice signal, and if applicable, the CC extraction techniques used; the normalization strategy used; the number of feature vector coordinates after the dimensionality reduction process; the use or nonuse of a data balancing technique; and the employed multiprojection techniques, if applicable. It is worth noting that all this information is split by the “|” identifier. For example, when analyzing the ACC metric for the case in which we consider only the voices of male individuals uttering the vowel \a\ in the low tonality, we have the following result: “100.0|CQCC, LFCC|Robust|24 |SVM|SMOTE|SUM, STD”, which means that the configuration of the proposed method that obtained 100% of accuracy makes use of CC extraction techniques based on CQCC and LFCC (P={CQCC,LFCC}) and does not use any noncepstral features (N={}); uses robust normalization ($\mathrm{NORM}={\mathrm{NORM}}_{\mathrm{ROB}}$); reduces the feature vector to 24 coordinates;uses SVM as classifier; uses the SMOTE balancing strategy; and performs multiprojection with the SUM and STD mapping functions ($M=\{m_{\mathrm{SUM}},m_{\mathrm{STD}}\}$). Observing the results displayed in Table 4, some interesting facts can be checked, as highlighted in the following paragraphs.

The proposed material was able to obtain maximum accuracy (100%) for all cases of the analyzed male voices. That is, regardless of the vowel and speech intonation, the method was able to correctly detect the presence or absence of dysphonia in all the male voice signals. Consequently, the EER and F1 values are also the best possible values for this voice base clipping. On the other hand, if we analyze the values referring to the average accuracy of the 5-fold cross-validation, or the KFCV, we realize that the method was not as easy to classify for male individuals. In fact, according to this metric, the method obtained better values when restricted to female individuals. This serves as an indication of the fact that the division between training and testing may have possibly favored the representation of male voices, while general random divisions, as is the case of the procedure considered for the calculation of the KFCV metric, may result in a greater difficulty in representing the voice signal, and consequently, the performance of the method may be compromised. However, even under these conditions, the proposed method was able to present KFCV values between 83% in the case of the high-pitched vowel \u\ and 95% in the case of the vowel \a\ with the intonation low–high–low, which are reasonably high values for this metric.

We can observe that for the clipping referring to female voices, the proposed method was also able to obtain maximum accuracy in most of the analyzed cases, having presented 95.35% as the lowest value for the case of the vowel \u\ uttered in a low tone. A similar analysis can be noted with respect to the other metrics, highlighting the case of the KFCV values, in which the method presented values between 95% and 97%. Consequently, we noticed that the clipping restricted to female voices is more representative, since the division between training and testing does not seem to drastically affect the performance of the technique. However, this selection is unbalanced, and in all situations for the KFCV metric, the analyzed configuration was only able to reach the best value using SMOTE, which indicates that the artificial balancing was effective.

As expected for this type of problem, the worst values of most metrics are associated with the database in which the voices of individuals of both genders are considered together. Specifically, with respect to the ACC, F1, and EER metrics, the values presented in this case are worse than those presented with respect to the female and male voices. For example, the average between false positives and false negatives (EER) presented by the proposed method when we consider voices of both genders is between 5.88%, which is the best case that occurs when the vowel is \a\ and the tone is low–high–low, and 19.26%, which is the worst case that occurs when the vowel is \u\ and the tone is high. For the case of the KFCV metric, we noticed that the results are intermediate to the cases referring to each specific gender, which indicates that in general, the presence of male voices makes it more difficult to characterize the voice signals, and consequently more difficult to detect dysphonia, since we have higher KFCV values when we observe only the female voices and we have lower KFCV values when we only observe the male voices. Still, the values of KFCV presented by the configurations of the proposed method are relatively high, since they are between 89% and 92%. Thus, as the KFCV values are higher than the ACC values, we can also infer that there is a possibility that the division between training and testing chosen for the voice database has not been advantageous for the proposed method when we consider voices of both genders.

Even though the selection generated by the Saarbruecken voice base is not necessarily the same as that of the other works discussed in Section 2, bearing in mind that the proposed method was able to present maximum accuracy in most of the cases in which we restricted the analysis of a given gender and that presented high accuracy for the selection in which we considered both genders, we can infer that the proposed framework is competitive in relation to the methods that make up the state of the art, according to Table 1.

**Figure 5:**
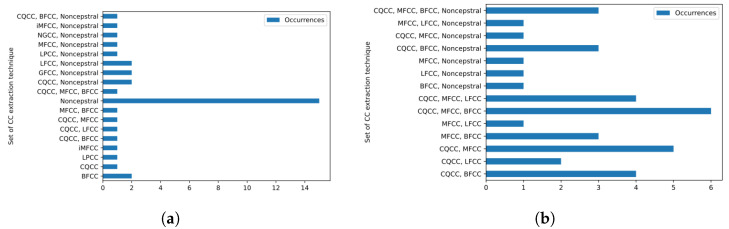
Histogram representing the number of configurations that used each indicated technique to obtain the best metric value in each case considering gender, vowel, and intonation; (**a**) EER metric; (**b**) KFCV metric.

When we analyze metrics that are directly associated with the division of training and testing that we employ in the voice database—which is the case of the ACC, F1, and ERR metrics—we realize that just using noncepstral features is enough to obtain the best metric value for some analyzed cases. For example, we can see that for the case referring to female voices, for all vowels uttered in the low tone, only noncepstral features were used in the configuration that presented the lowest EER. However, this does not occur when we analyze metrics associated with more generalized cases, as is the case of KFCV. For this metric, no configuration was able to reach the maximum value using only noncepstral features. In fact, all configurations make use of at least two CC extraction techniques to compose the feature vector, being the configuration that considers P={CQCC,MFCC,BFCC} to be the one that performs best most of the cases. In Figure 5, we present a histogram that represents the number of configurations that obtained the best value for EER (Figure 5a) and KFCV (Figure 5b) metrics in each case referring to gender, vowel and tone. We can see that the isolated use of noncepstral features is the most common for the EER case. However, in most cases, at least one cepstral feature was used to obtain the best EER value.

**Figure 6:**
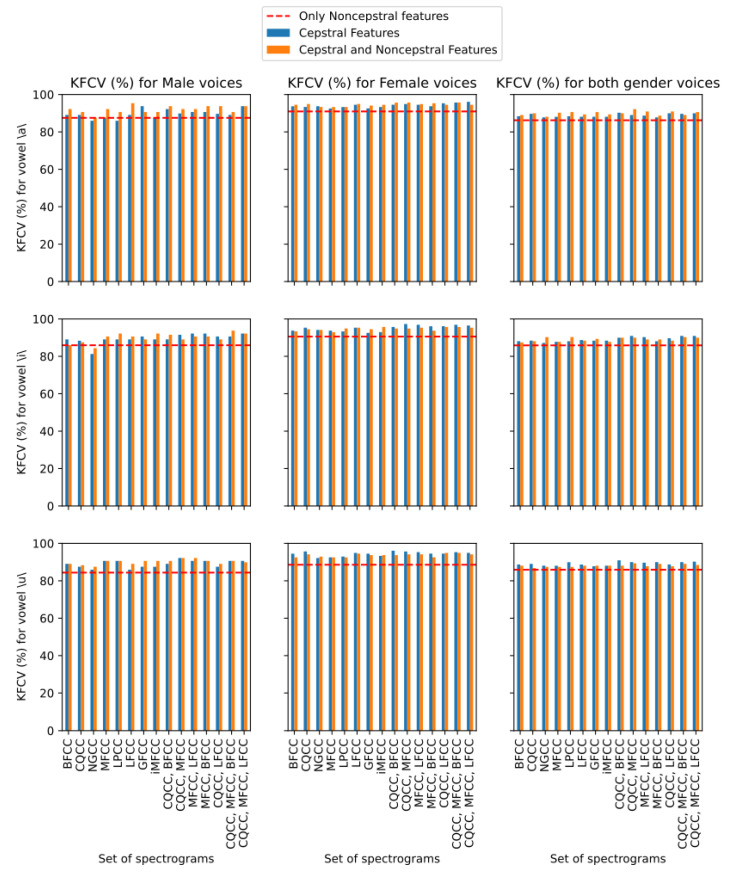
Comparison of the impact of using cepstral and noncepstral features according to the KFCV metric. The results are presented according to the gender of the analyzed voices and according to each vowel. Furthermore, only the best value among all analyzed configurations for each combination of CC extraction techniques was presented.

We can analyze in greater detail the performance improvement of the technique with the use of cepstral features from Figure 6, which shows the best KFCV metric obtained by each combination of cepstral features, or P, considered in this work with and without the aggregate use of noncepstral features, represented, respectively, by the orange and blue bars. Also represented by a red dashed line is the KFCV metric obtained by the single use of noncepstral features (N). For each technique, the highest value of KFCV obtained between the intonations of each vowel is presented, and the results are presented by vowel and by voice gender. Immediately, we can see that there is a strong tendency for the use of any cepstral feature to be superior to the isolated use of noncepstral features, since in all cases, most of the blue and orange bars are above the dashed red line. In fact, only a few combinations of P perform worse than the isolated use of noncepstral features when we look at the results for male voices with respect to the vowels \a\ and \i\. Thus, we can infer that the use of cepstral features brings more robustness to the classification model proposed in the voice dysphonia detection framework. In addition, we can also note that the bars referring to the joint use of more than one CC extraction technique lead to higher KFCV values, since the rightmost bars of each chart tend to be larger than the leftmost bars. We can also observe a difference in the efficiency of the combination of cepstral and noncepstral features for each observed gender, since, for male voices, we noticed a slight tendency for the orange bars to be above the blue bars; that is, the cepstral and non-cepstral features are more representative than just the cepstral ones, while for female voices, this trend is very poorly defined, as we have many cases in which the blue bars are greater than the orange bars, and vice versa. Furthermore, this pattern propagates to the case where voices of both genders are considered. This phenomenon may be associated with the fact that male voices are more difficult to analyze in terms of dysphonia detection, and consequently, the use of features that represent patterns of different natures seems to be more effective in this process for this gender.

**Figure 7:**
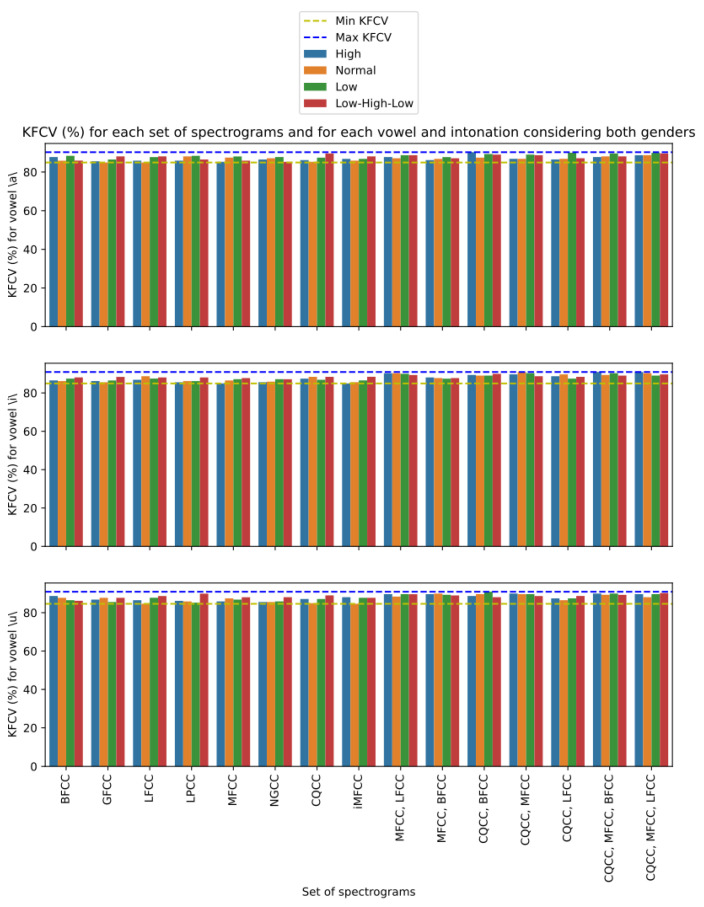
Comparison of the impact of using more than one cepstral feature at the same time according to the KFCV metric for voices of both genders. For each vowel, a bar graph was made, in which each bar represents the performance of a technique with respect to each intonation. It is worth mentioning that only the results of the analyzed versions with cepstral features are shown in this figure. In other words, for all cases analyzed here, N={}. Furthermore, only the best value among all analyzed configurations for each combination of CC extraction techniques was presented.

Still on the joint use of more than one CC extraction technique, we present in Figure 7 the performance of each combination of P with respect to KFCV, considering only cepstral features, that is, N={}, for voices of both genders. For each vowel, a bar graph was made, in which each bar represents the performance of the analyzed version on each intonation of the respective vowel, in which the intonations of high, normal, low, and low–high–low are represented, respectively, by the colors blue, orange, green, and red. The lowest and highest values of KFCV in each vowel are also highlighted, represented, respectively, by the yellow and blue dashed lines. With respect to all vowels, we can see a trend that the bars on the right are higher, and consequently are closer to the maximum value of KFCV, while the bars on the left are closer to the minimum value of KFCV. This indicates that proposed material configurations that make use of more than one CC extraction technique tend to perform more favorably compared to configurations that use only one of these techniques. We can also observe that this pattern is not restricted to a certain vowel or a certain intonation, since this pattern is replicated in all analyzed situations. However, we can note that some CC extraction techniques are efficient in isolated situations. For example, we can see that the LPCC technique showed a high KFCV value for the vowel \u\ with low–high–low intonation, and that the CQCC technique showed a high KFCV value for the vowel \a\ with the intonation low–high–low. However, it is a good performance that occurs occasionally, unlike the stable performance shown by settings such as P={CQCC,MFCC,BFCC} or P={CQCC,MFCC,LFCC} that obtained high KFCV values for all vowels in most intonations.

**Figure 8:**
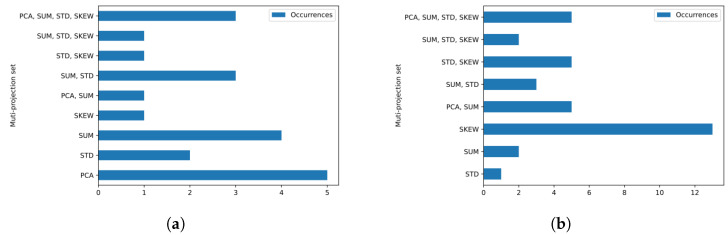
Histogram representing the number of configurations that used each multiprojection strategy to obtain the best metric value in each case considering gender, vowel, and intonation; (**a**) EER metric; (**b**) KFCV metric.

Analyzing the configurations in which at least one cepstral feature was used to obtain the best metric, we also noticed some trends. For example, in the case of the EER metric, the use of the mapping function based on the PCA projection on the CCs was the most common strategy in the configurations that obtained lower EER through the use of cepstral features. In fact, in most configurations, using only one projection function was enough to obtain the lowest EER. On the other hand, in the case of the KFCV, which represents the most generalized training and test division approach on the database, we can see that although the use of the mapping function based on the skewness of the CCs was the most common function among the configurations, most configurations made use of more than one mapping function, which indicates that in general, the use of more than one mapping function, which defines the concept of multiprojection, is superior in representing the CCs for the problem detection of voice dysphonia. In Figure 8, we present the histogram that counts the number of configurations based on CCs that obtained the best metrics for EER (Figure 8a) and for KFCV (Figure 8b) according to each multiprojection set.

**Figure 9:**
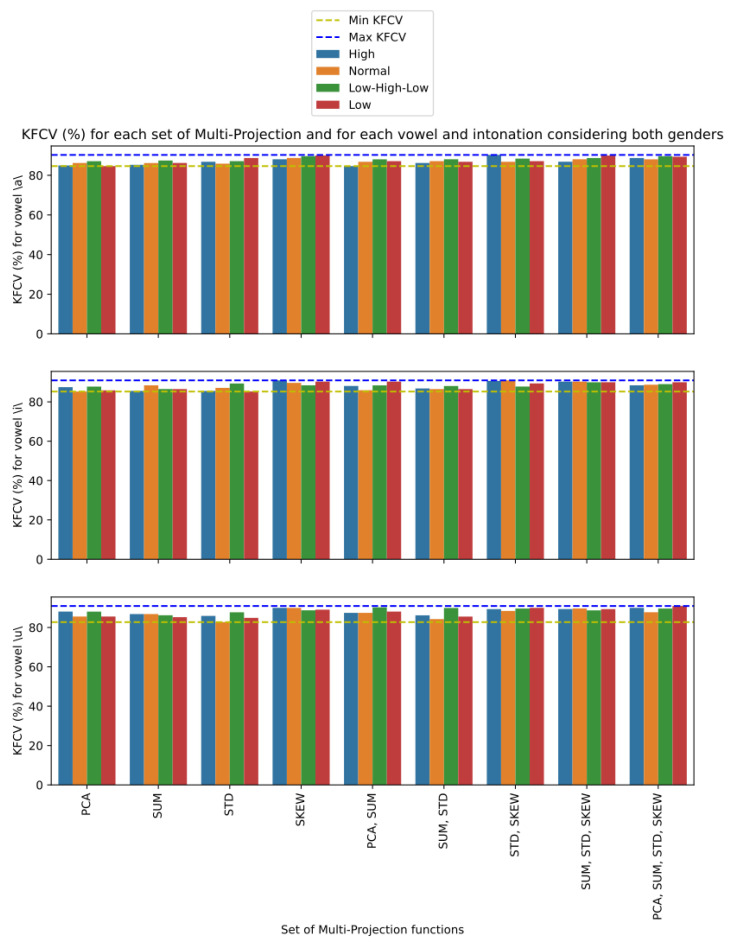
Comparison of the impact of using more than one mapping function on one or more cepstral features according to the KFCV metric for voices of both genders. For each vowel, a bar graph was made, in which each bar represents the performance of a certain technique with respect to each intonation. It is worth mentioning that only the results of the analyzed versions with cepstral features are shown in this figure. In other words, for all cases analyzed here, N={}. Furthermore, only the best value among all analyzed configurations for each combination of mapping functions was presented.

To carry out a detailing, we present in Figure 9 the best performance results according to the KFCV metric restricted to the projection techniques used on the cepstral features of each configuration that are defined on this feature class. Furthermore, only configurations that do not use noncepstral features were considered, i.e., N={} in this case. The presented results refer to the voices of individuals of both genders and are divided according to vowels, and a bar graph was made referring to each one, and according to the intonations. Analogously to the analyses referring to the use of more than one CC extraction technique to represent the voice signal, we noticed that the use of more than one mapping function to project the CCs also seemed to be associated with higher KFCV values. In fact, except the mapping function $m_{\mathrm{SKEW}}$, which presents stable high KFCV results, the other mapping functions employed in isolation present the lowest KFCV values for all intonations of all vowels, reserving the highest and most stable values for configurations that make use of at least three mapping functions. However, we noticed that all combinations of functions that present high KFCV values for all cases use the $m_{\mathrm{SKEW}}$ function, which is already a function associated with high KFCV values. So, we can infer that the other mapping functions enrich the good representation capacity of $m_{\mathrm{SKEW}}$, and consequently, indicate that the use of more than one mapping function can serve to bring about stability and improve the results of mappings that are already effective in the analyzed problem.

**Figure 10:**
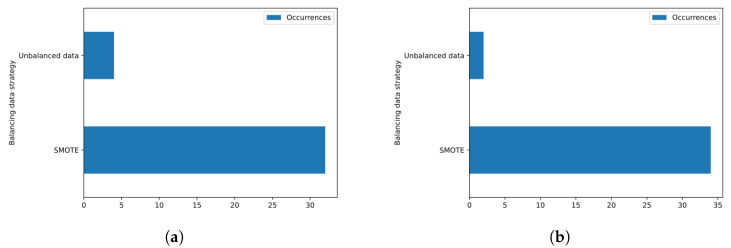
Histogram representing the number of configurations that made use of the SMOTE balancing strategy to obtain the best metric value in each case considering gender, vowel and intonation; (**a**) EER metric; (**b**) KFCV metric.

The selection obtained by the Saarbruecken voice database is slightly unbalanced for the case of samples from male individuals, with a proportion of 54.24% of healthy samples and 45.76% of dysphonic samples, and severely unbalanced for the case of samples from female subjects, with a ratio of 75.59% of healthy samples and 24.41% of dysphonic samples. Because of this, in most analyzed cases, the best configuration made use of the SMOTE balancing technique. In particular, we noticed that for the KFCV metric, in all cases, the configurations that obtained the best performance on the selection referring to the female gender required the use of balancing, while for the selection referring to the male samples for the vowels \a\ and \i\ and the high intonation, the best-performing settings did not require balancing. However, as we can see in the histograms in Figure 10, in most of the analyzed cases, balancing was necessary for the best-performing configurations according to the EER (Figure 10a) and KFCV (Figure 10b) metrics.

**Figure 11:**
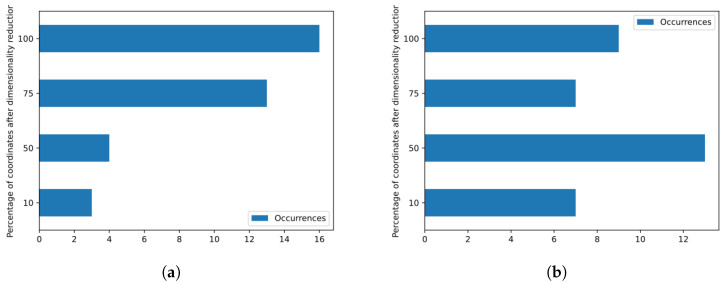
Histogram representing the percentage of coordinates of the feature vectors of the best configurations according to the EER and KFCV metrics in each case considering gender, vowel, and intonation. In this case, 100% means that there was no dimensionality reduction; (**a**) EER metric; (**b**) KFCV metric.

The adopted dimensionality reduction strategy proved to be effective in most of the analyzed cases. As detailed in Figure 11, the use of 100% of the feature vector coordinates, i.e., the use of all original feature vector coordinates, was associated with the best configuration for the EER metric in 16 cases and for the KFCV metric in 9 cases, while in the other cases, the dimensionality reduction was associated with the configurations that obtained the best performance values. In addition, we can see that for the division into training and testing adopted on the voice database, the analyzed configurations required a higher percentage of coordinates in the feature vector to obtain the best EER value, since the most common values in the histogram of Figure 11a are 100% and 75%, totaling 29 cases. On the other hand, when we observe random variations of training and testing, which is what happens when we compute the value of KFCV, most configurations with better performance use only 50% of the total number of coordinates, as can be seen in Figure 11b.

**Figure 12:**
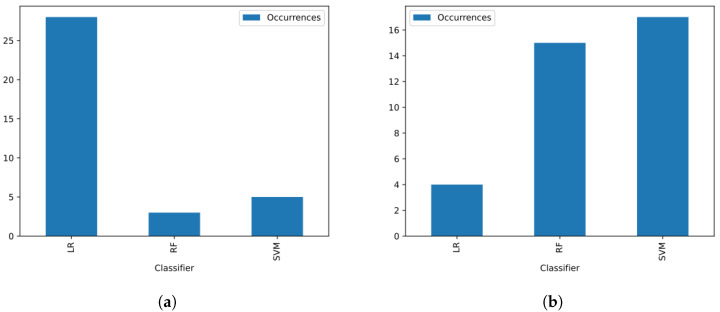
Histogram representing the number of times each classifier composed the best configuration according to the EER and KFCV metrics in each case considering gender, vowel, and intonation; (**a**) EER metric; (**b**) KFCV metric.

Among the three classifiers considered in the configurations of the proposed material, we noticed when analyzing Figure 12a a strong tendency of the LR classifier composing most of the versions that presented the best EER result. This pattern makes it clear that the division into training and test sets made on the voice database is a division that does not represent the general case, since in most of the works discussed in Section 2, the SVM and RF classifiers are employed with a higher success rate in solving the voice dysphonia detection problem. Furthermore, when analyzing the KFCV metric in Figure 12b, we noticed that these two classifiers become the majority among the configurations with the best values for this metric. In particular, the well-known SVM is the most used classifier in the topic addressed, and proved to be the most efficient in most cases, according to the KFCV.

**Figure 13:**
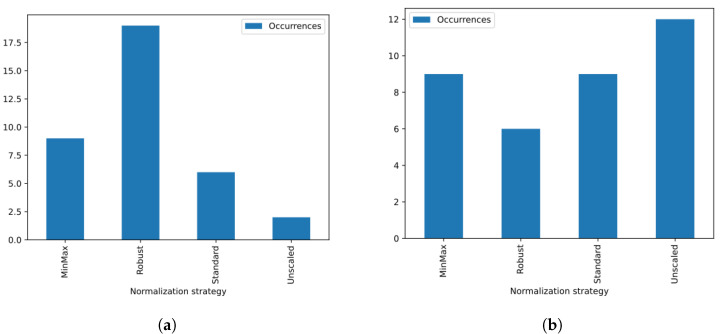
Histogram representing the number of times each normalization strategy composed the best configuration according to the EER and KFCV metrics in each case considering gender, vowel, and intonation; (**a**) EER metric; (**b**) KFCV metric.

Finally, when analyzing the occurrences in which each normalization strategy was present in the composition of the configuration associated with the best EER and KFCV values, as highlighted in the histograms in Figure 13, we noticed that according to both metrics, most of these configurations made use of some normalization strategy. For the case of the EER metric, we noticed that robust normalization was the most common, which indicates that the division between training and testing made on the voice database may have counted on the presence of outliers. While, in general, when analyzing the KFCV metric, we noticed that even though most of the configurations made use of some normalization strategy, not adopting a normalization strategy was the most common procedure among the configurations.

### 7.3. Complexity Analysis

We can observe that the complexity of the proposed framework is dependent on the techniques that make up its foundation. In other words, our material has variable complexity according to the parameterization considered for the sets of techniques for extracting CCs (P), mappings (M), and techniques for extracting noncepstral features (N). Specifically, the complexity of the proposed method is compounded by the accumulated complexity to compute the cepstral features, the projection of these features, and the noncepstral features. Mathematically, the complexity of the method is formed by the complexity of all the techniques $\Phi_{i}\in P$ and by the complexity of calculating their respective first- and second-order derivatives, which are complexities of the order of $O\left(n_{\mathrm{ceps}}\cdot n_{\mathrm{frames}}\right)$ for the case that the CCs are given according to Equation (1), together with the complexities of all the M mapping functions used to map each of the $n_{P}$ CCs and each of their derivatives, plus the complexities of N techniques. Therefore, the complexity of the proposed method is given by $O_{\mathrm{Proposed}}$ defined in Equation (10):

(10) $$O_{\mathrm{Proposed}}=\sum_{i=1}^{n_{P}}\left[O_{\Phi_{i}}+2\cdot O\left(n_{\mathrm{ceps}}\cdot n_{\mathrm{frames}}\right)\right]+3n_{P}\sum_{i=1}^{n_{M}}O_{m_{i}}+\sum_{i=1}^{n_{N}}O_{\mu_{i}},$$

where $O_{\Phi_{i}}$ is the complexity of the technique $\Phi_{i}$, $\forall \Phi_{i}\in P$; $O_{m_{i}}$ is the complexity of the mapping $m_{i}\in M$, $\forall m_{i}\in M$; and $O_{\mu_{i}}$ is the complexity of the technique $\mu_{i}$, $\forall \mu_{i}\in N$.

It is worth mentioning that in this work, we are considering static cepstral features; in this case, the CCs and dynamic ones, which are Δ and ΔΔ. If we do not consider the dynamic cepstral features, which is relatively common in the dysphonia detection problem, the complexity of the proposed material would be reduced, as we would not have the complexities of calculating these matrices; that is, we would not need to consider $2O\left(n_{\mathrm{ceps}}\cdot n_{\mathrm{frames}}\right)$ for each technique from P. Furthermore, we would not need to project these features, and consequently, the complexity accumulated by the projections would be reduced from $3n_{P}\sum_{i=1}^{n_{M}}O_{m_{i}}$ to $n_{P}\sum_{i=1}^{n_{M}}O_{m_{i}}$.

**Figure 14:**
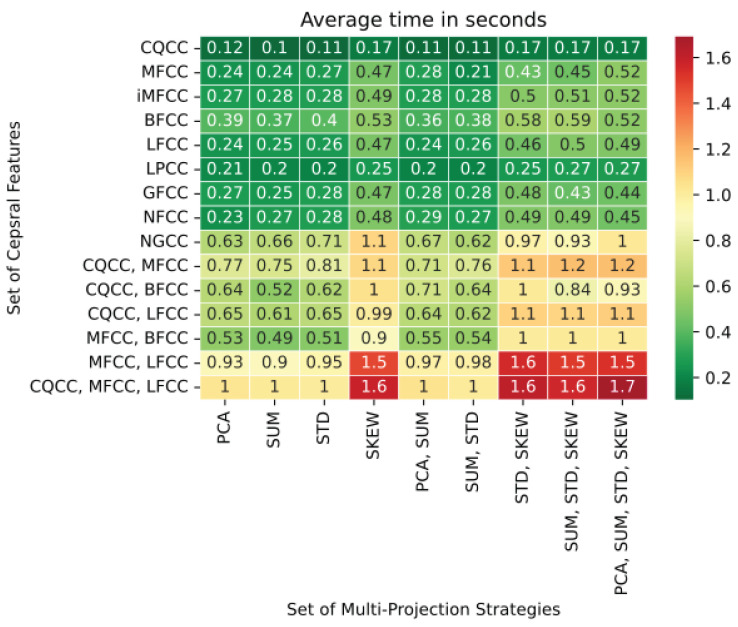
Average time in seconds for feature extraction from a sound signal considering each CC extraction technique with respect to each multiprojection strategy. Values have been rounded to present a maximum of two decimal places.

In our experiments, we computed the average feature extraction and representation time for the CC calculation techniques considered in P and with respect to the mapping functions of M. Figure 14 shows an average time, in seconds, that the proposed method takes to represent a voice signal using each configuration evaluated in P and in M. For example, to represent a voice signal with the CCs extracted by the CQCC technique and projected by PCA, the proposed method took, on average, 0.12 s. The chart does not consider the extraction time of noncepstral features, referring to the portions $\sum_{i=1}^{n_{N}}O_{\mu_{i}}$ of Equation (10), since this amount, which is approximately 0.78 s, is the same value in all cases.

When analyzing the time used by the method according to its configurations, we can notice that there is a close correlation with the complexity defined in Equation (10), since the configurations that use less computational time are those that make use of only one cepstral feature and only one projection function. In addition, the configurations that consume the most resources are those that use more cepstral features—with emphasis on the configurations defined by the MFCC and LFCC techniques and by the CQCC, MFCC, and LFCC techniques—and more than one projection function. We also realized that the projection by the skewness measure entails more computational time when compared to the other mappings. In fact, the joint mappings formed by functions $m_{\mathrm{PCA}}$ and $m_{\mathrm{SUM}}$ and by functions $m_{\mathrm{SUM}}$ and $m_{\mathrm{STD}}$ do not entail, in practice, more computational time compared to its isolated use. Thus, we note that what contributes to a considerable increase in computational time are the cepstral features, once the use of multiprojection does not perceptibly change the computational time in the proposed method.

### 7.4. Limitations

For the proper execution of the proposed method, it is necessary to configure a large number of parameters, since it is defined in a generalized way. As discussed in this section, there are many variations of the method that can be configured, but we need to look at them together to obtain the most accurate results. This implies a series of experimentation steps that can be quite extensive and time-consuming.

The proposed framework requires a series of processes to be performed to increase classification accuracy. These processes include the representation of sound signals through sets of CC extraction techniques, the realization of multiprojections of these CCs, the data balancing process, the dimensionality reduction, the normalization of the feature vectors, and the training of classifiers. All these processes are essential for the effectiveness of the framework, but they require processing time. Therefore, it is essential that these processess are properly configured to ensure that the voice dysphonia detection system does not slow down and can function optimally.

## 8. Conclusions

In this work, two significant advances were presented to improve security in BASs through the detection of dysphonia in voice signals. The first advance focused on the generalization of the vector representation of cepstral features, which are originally given in matrices, through the use of sets of mapping functions. This multiprojection process allowed for a more accurate and efficient representation of sound signals, improving dysphonia detection. The second advance consisted of the creation of a framework that allowed for the use of these multidesigned cepstral features in conjunction with other noncepstral features. This made it possible to increase the representation capacity of the sound signal, and consequently, to improve the accuracy of the classifiers. This framework involved performing several processes, such as the representation of sound signals through sets of CC extraction techniques, performing multiprojections of these CCs, the data balancing process, dimensionality reduction, and normalization of feature vectors, in addition to training classifiers. These processes were critical to the effectiveness of the framework and required careful analysis to ensure accurate and reliable results.

The work presented important contributions in general, but it was necessary to define practical instances of the material to conduct evaluations on its functioning. For this, eight CC extraction techniques were used, along with the proposed multiprojection representation strategy. These techniques were employed to establish fifteen different combinations of cepstral features in P in the proposed framework. Experiments were carried out with all practical instances defined and compared among themselves according to their results on the Saarbruecken voice base. Several metrics were analyzed to evaluate the performance of the proposed framework. Metrics that acted on a specific division of training and testing of the selection of the Saarbruecken base were considered, such as the metrics ACC, EER, and F1; as well as metrics that acted on multiple dynamically and randomly defined divisions on the selection of the base, such as the KFCV metric. The results showed that the proposal presented competitive numerical values in relation to the state of the art regarding the considered metrics. In addition, it was possible to observe the influence of specific stages of the proposed framework, which had their effectiveness proven by the results obtained in the analyzed metrics. These results confirm the effectiveness of the proposed framework and provide a solid basis for its use in BASs. It was also possible to verify that the processes of fusion of cepstral features and the use of multiprojection strategies with more than one mapping function proved to be efficient in the representation of the voice signal, and consequently, in the routine of detecting voice dysphonia.

For future experiments, it is recommended to use more elaborate strategies to compose the steps of the proposed framework, since until now, only simple strategies have been considered. Even so, the results obtained were satisfactory. For example, the use of deep learning networks could be used in three stages of the framework: as one of the projection functions in M, responsible for nonlinearly extracting the cepstral features of the voice signal; as a dimensionality reduction technique, reducing the redundancy contained in the vector ${\vec{v}}_{x}$; or as the model classifier. Furthermore, it is possible that the proposal presents equally good results in problems of detecting other abnormalities in the voice, such as other pathologies available in the Saarbruecken voice base or in other voice bases. Finally, it is important to evaluate the proposal in other voice pattern recognition problems, such as the spoofing detection problem, which also has little experimentation with respect to the multiprojection strategies of cepstral features.

## Figures and Tables

**Table 1:** Comparison between the main studies on voice disorder detection addressed in this section. Accuracy is given as a percentage. The symbol “-”  means that the information is not available in the cited work. The meanings of abbreviations in the text that are not yet defined, as well as those already defined, can be found in the final section of this work.

Work	Features	Reduction	Classifier	Database	Accuracy	Year
[53]	Jitter, shimmer, $F_{0}$ metrics	All possible combinations of features are considered	LDA	MEEI	96.5	2001
[69]	MFCC	-	MLP, LVQ	MEEI	96	2004
[70]	MFCC, modulation spectral features	Singular value decomposition	GMM, SVM	MEEI, UPM	95.89	2011
[62]	Energy-based features of GA-adaptive wavelet	-	SVM	MEEI	100	2013
[54]	Jitter, shimmer, HNR	PCA	ANN	SVD	100	2017
[60]	DWT, ILPF	-	-	USPD	85.94	2017
[71]	MFCCs, line spectral frequencies	-	GMM, SVM, discriminant analysis	MEEI	98.7	2017
[32]	SWT-based energy and statistical features	Information gain	SVM, SGD, ANN	tSVD, PdA, AVPD, MEEI	99.99	2020
[56]	Energy, entropy, ZCR	Paraconsistent engineering	Paraconsistent discrimination, SVM	SVD	95	2020
[61]	HOS, EMD-DWT	-	SVM	SVD	99.26	2020
[63]	DTCWT - Tsallis entropy	-	KNN, SVM	MEEI, SVD	93.32	2020
[37]	MFCC, LPCC, HOS	-	CNN, FNN	SVD	82.69	2021
[52]	Pitch-based	Fisher discriminant ratio, stepwise discriminant analysis, scatter measure, ANOVA statistical test, divergence measure, relief F-measure, run filtering	KNN, SVM, RF, NB	DPV	91.5	2022
[31]	SWT-based energy and statistical features	Probability density plots, information gain	SVM, SGD	tSVD, PdA, AVPD, MEEI	99.99	2022
[72]	MFCC, Mel-spectrogram, chroma	-	DNN	SVD	96.77	2022
[55]	Autocorrelation, HNR, NHR	-	-	SVD, USPD	-	2023
[73]	MFCC, LPCC	-	CNN, FNN	SVD	98.89	2023
Proposed	$F_{0}$, HNR, jitters and shimmers variations, formant frequencies measures, CQCC, MFCC, iMFCC, BFCC, LFCC, LPCC, GFCC, NGCC	Singular value decomposition	SVM, RF, LR	SVD	100	2023

**Table 2:** Considered configurations for the sets of mappings used in the experiments.

Set	Mapping Functions
$M_{1}$	$m_{P}CA$
$M_{2}$	$m_{S}UM$
$M_{3}$	$m_{S}TD$
$M_{4}$	$m_{S}KEW$
$M_{5}$	$m_{P}CA$, $m_{S}UM$
$M_{6}$	$m_{S}UM$, $m_{S}TD$
$M_{7}$	$m_{S}TD$, $m_{S}KEW$
$M_{8}$	$m_{S}UM$, $m_{S}TD$, $m_{S}KEW$
$M_{9}$	$m_{P}CA$, $m_{S}UM$, $m_{S}TD$, $m_{S}KEW$

**Table 3:** Considered configurations for the sets of CC extraction techniques used in the experiments.

Set	CC Extraction Technique
$P_{1}$	CQCC
$P_{2}$	MFCC
$P_{3}$	iMFCC
$P_{4}$	BFCC
$P_{5}$	LFCC
$P_{6}$	LPCC
$P_{7}$	GFCC
$P_{8}$	NGCC
$P_{9}$	CQCC, MFCC
$P_{10}$	CQCC, BFCC
$P_{11}$	CQCC, LFCC
$P_{12}$	MFCC, BFCC
$P_{13}$	MFCC, LFCC
$P_{14}$	CQCC, MFCC, LFCC
$P_{15}$	CQCC, MFCC, BFCC

**Table 4:** Summary of the performance results of all analyzed configurations of the proposed method. Results are displayed for the metrics ACC (%), KFCV, F1, and EER (%). Only the best result obtained among all method configurations is presented in each case, and together with the result, details of the respective confirmation are highlighted. In detail, the term “nonceps” indicates the use of noncepstral features described in the set N, and which define the vector ${\vec{v}}_{x,\mathrm{non}-\mathrm{cepstral}}$. If this term does not appear in the description of the techniques, then only cepstral features were used. Furthermore, in case the best configuration uses cepstral features, or the vector ${\vec{v}}_{x,\mathrm{cepstral}}$, we also highlight the multiprojection strategy employed. Furthermore, the use of balancing technique is indicated by the word SMOTE, while nonuse is indicated by the symbol “-”. The results are partitioned by evaluated metric, gender of the individuals that make up the voice base, and by the combination of vowels and intonation, or vowel\pitch (V\P).

Metric		ACC (%)			
Gender	V\P	High	Low	Low-High-Low	Normal
Both	a	91.23|nonceps|Robust|23|LR|SMOTE	92.98|nonceps|Standard|31|LR|-	96.49|nonceps|Standard|31|LR|SMOTE	94.74|nonceps|Standard|31|LR|-
	i	85.96|MFCC, nonceps|Robust|91|LR|-|STD	91.23|NGCC, nonceps|Robust|91|LR|SMOTE|STD	85.96|BFCC|Standard|120|SVM|SMOTE|SUM, STD	87.72|GFCC, nonceps|Robust|91|LR|-|STD
	u	85.96|LFCC, nonceps|Robust|91|RF|SMOTE|PCA	92.98|nonceps|Robust|31|LR|SMOTE	87.72|GFCC, nonceps|MinMax|46|LR|-|STD	89.47|iMFCC, nonceps|Standard|91|LR|SMOTE|PCA
Female	a	97.67|iMFCC, nonceps|MinMax|68|LR|-|PCA	100.0|nonceps|Standard|23|LR|SMOTE	100.0|nonceps|Robust|23|LR|SMOTE	100.0|nonceps|Standard|23|LR|SMOTE
	i	100.0|iMFCC, nonceps|Standard|68|LR|SMOTE|SUM	100.0|nonceps|MinMax|23|SVM|SMOTE	100.0|CQCC, nonceps|Robust|68|LR|-|SUM	100.0|LFCC, nonceps|Robust|68|LR|SMOTE|SUM
	u	97.67|nonceps|Standard|23|LR|-	95.35|nonceps|MinMax|23|SVM|-	100.0|iMFCC, nonceps|MinMax|68|LR|SMOTE|PCA	100.0|nonceps|Standard|23|LR|SMOTE
Male	a	100.0|LFCC, nonceps|MinMax|15|SVM|SMOTE|SUM, STD	100.0|nonceps|Standard|31|LR|SMOTE	100.0|nonceps|Robust|31|LR|-	100.0|nonceps|Standard|16|LR|SMOTE
	i	100.0|CQCC, MFCC, BFCC|Robust|720|LR|SMOTE|PCA, SUM, STD, SKEW	100.0|MFCC, BFCC|Robust|120|RF|SMOTE|STD	100.0|CQCC, LFCC|Robust|240|LR|SMOTE|STD, SKEW	100.0|CQCC, BFCC|Unscaled|180|RF|SMOTE|PCA, SUM
	u	100.0|CQCC, LFCC|Robust|24|SVM|SMOTE|SUM, STD	100.0|NGCC, nonceps|Unscaled|46|RF|SMOTE|SKEW	100.0|iMFCC|Robust|30|LR|SMOTE|PCA	100.0|CQCC, MFCC|Standard|480|LR|SMOTE|PCA, SUM, STD, SKEW
Metric		KFCV			
Gender	V\P	High	Low	Low-High-Low	Normal
Both	a	0.90|CQCC, BFCC|MinMax|240|RF|SMOTE|STD, SKEW	0.90|CQCC, LFCC|Standard|12|RF|SMOTE|SKEW	0.92|CQCC, MFCC, nonceps|Unscaled|136|LR|SMOTE|PCA, SUM	0.89|CQCC, MFCC, LFCC|Unscaled|135|SVM|SMOTE|SKEW
	i	0.91|CQCC, MFCC, BFCC|Standard|180|SVM|SMOTE|SKEW	0.90|CQCC, MFCC, BFCC|Robust|36|RF|SMOTE|PCA, SUM	0.90|CQCC, BFCC|Robust|36|RF|SMOTE|SUM, STD, SKEW	0.91|CQCC, MFCC|Standard|180|SVM|SMOTE|STD, SKEW
	u	0.90|CQCC, MFCC, BFCC|Unscaled|90|SVM|SMOTE|SKEW	0.91|CQCC, BFCC|Robust|48|SVM|SMOTE|PCA, SUM, STD, SKEW	0.90|CQCC, MFCC, LFCC|Unscaled|180|RF|SMOTE|PCA, SUM	0.90|CQCC, MFCC|Unscaled|270|SVM|SMOTE|SUM, STD, SKEW
Female	a	0.96|CQCC, MFCC, LFCC|Robust|270|SVM|SMOTE|STD, SKEW	0.95|MFCC, BFCC, nonceps|Robust|136|RF|SMOTE|PCA, SUM	0.96|CQCC, MFCC, LFCC|Robust|180|SVM|SMOTE|SKEW	0.95|CQCC, MFCC|Robust|120|SVM|SMOTE|SKEW
	i	0.97|CQCC, MFCC, BFCC|MinMax|180|SVM|SMOTE|SKEW	0.96|CQCC, MFCC, BFCC|Robust|360|SVM|SMOTE|PCA, SUM, STD, SKEW	0.96|CQCC, MFCC, LFCC|MinMax|180|SVM|SMOTE|SKEW	0.97|CQCC, MFCC|MinMax|240|SVM|SMOTE|STD, SKEW
	u	0.95|CQCC, MFCC, BFCC|Standard|360|SVM|SMOTE|PCA, SUM, STD, SKEW	0.96|CQCC, BFCC|Standard|60|SVM|SMOTE|SKEW	0.96|CQCC, BFCC|Robust|12|SVM|SMOTE|SKEW	0.95|CQCC, MFCC, BFCC|Unscaled|18|RF|SMOTE|SUM
Male	a	0.85|iMFCC, nonceps|Unscaled|68|RF|-|PCA	0.92|MFCC, LFCC, nonceps|Standard|76|LR|SMOTE|STD	0.95|LFCC, nonceps|Standard|15|RF|SMOTE|STD, SKEW	0.88|MFCC, BFCC, nonceps|Robust|256|LR|SMOTE|PCA, SUM, STD, SKEW
	i	0.92|CQCC, BFCC, nonceps|Unscaled|136|RF|-|SUM, STD	0.88|MFCC, BFCC|Unscaled|240|RF|SMOTE|PCA, SUM	0.94|CQCC, MFCC, BFCC, nonceps|Unscaled|39|RF|SMOTE|PCA, SUM	0.84|CQCC, MFCC, LFCC, nonceps|Robust|563|RF|SMOTE|PCA, SUM, STD, SKEW
	u	0.83|CQCC, nonceps|Standard|91|RF|SMOTE|SUM	0.89|MFCC, nonceps|Robust|9|LR|SMOTE|SUM	0.92|CQCC, MFCC, nonceps|Standard|271|RF|SMOTE|SUM, STD	0.86|CQCC, MFCC, BFCC|Unscaled|270|RF|SMOTE|SUM, STD
Metric		F1			
Gender	V\P	High	Low	Low-High-Low	Normal
Both	a	0.94|nonceps|Robust|23|LR|SMOTE	0.95|nonceps|Standard|31|LR|SMOTE	0.98|nonceps|Standard|31|LR|SMOTE	0.96|nonceps|Standard|31|LR|-
	i	0.91|MFCC, nonceps|Robust|91|LR|-|STD	0.94|NGCC, nonceps|Robust|91|LR|SMOTE|STD	0.90|BFCC|Standard|120|SVM|SMOTE|SUM, STD	0.92|GFCC, nonceps|Robust|91|LR|-|STD
	u	0.91|LFCC, nonceps|Robust|91|RF|SMOTE|PCA	0.95|nonceps|Robust|31|LR|SMOTE	0.92|GFCC, nonceps|MinMax|46|LR|-|STD	0.93|iMFCC, nonceps|Standard|91|LR|SMOTE|PCA
Female	a	0.98|iMFCC, nonceps|MinMax|68|LR|-|PCA	1.00|nonceps|Standard|23|LR|SMOTE	1.00|nonceps|Robust|23|LR|SMOTE	1.00|nonceps|Standard|23|LR|SMOTE
	i	1.00|iMFCC, nonceps|Standard|68|LR|SMOTE|SUM	1.00|nonceps|MinMax|23|SVM|SMOTE	1.00|CQCC, nonceps|Robust|68|LR|-|SUM	1.00|LFCC, nonceps|Robust|68|LR|SMOTE|SUM
	u	0.98|nonceps|Standard|23|LR|-	0.97|nonceps|MinMax|23|SVM|-	1.00|iMFCC, nonceps|MinMax|68|LR|SMOTE|PCA	1.00|nonceps|Standard|23|LR|SMOTE
Male	a	1.00|LFCC, nonceps|MinMax|15|SVM|SMOTE|SUM, STD	1.00|nonceps|Standard|31|LR|SMOTE	1.00|nonceps|Robust|31|LR|-	1.00|nonceps|Standard|16|LR|SMOTE
	i	1.00|CQCC, MFCC, BFCC|Robust|720|LR|SMOTE|PCA, SUM, STD, SKEW	1.00|MFCC, BFCC|Robust|120|RF|SMOTE|STD	1.00|CQCC|Robust|120|LR|SMOTE|STD, SKEW	1.00|CQCC, BFCC|Unscaled|180|RF|SMOTE|PCA, SUM
	u	1.00|CQCC, LFCC|Robust|24|SVM|SMOTE|SUM, STD	1.00|NGCC, nonceps|Unscaled|46|RF|SMOTE|SKEW	1.00|iMFCC|Robust|30|LR|SMOTE|PCA	1.00|CQCC, MFCC|Standard|480|LR|SMOTE|PCA, SUM, STD, SKEW
Metric		EER (%)			
Gender	V\P	High	Low	Low-High-Low	Normal
Both	a	7.94|nonceps|Robust|23|LR|SMOTE	6.69|nonceps|Standard|31|LR|SMOTE	5.88|nonceps|Robust|31|LR|SMOTE	7.13|nonceps|Standard|31|LR|-
	i	18.82|BFCC|MinMax|120|SVM|SMOTE|SUM, STD	13.82|GFCC, nonceps|Robust|91|LR|SMOTE|SUM	14.19|LPCC|MinMax|60|SVM|-|PCA	18.82|LPCC, nonceps|Robust|91|LR|SMOTE|PCA
	u	19.26|BFCC|Standard|180|LR|SMOTE|SUM, STD, SKEW	11.76|nonceps|Robust|31|LR|SMOTE	16.76|CQCC, BFCC, nonceps|MinMax|51|LR|SMOTE|PCA, SUM, STD, SKEW	13.82|CQCC, nonceps|MinMax|46|LR|SMOTE|PCA
Female	a	1.56|nonceps|Robust|23|LR|SMOTE	0.00|nonceps|MinMax|23|LR|SMOTE	0.00|nonceps|Robust|23|LR|SMOTE	0.00|nonceps|MinMax|23|LR|SMOTE
	i	0.00|MFCC, nonceps|Standard|68|LR|SMOTE|SUM	0.00|nonceps|MinMax|23|SVM|SMOTE	0.00|CQCC, nonceps|Robust|68|LR|-|SUM	0.00|LFCC, nonceps|Robust|68|LR|SMOTE|SUM
	u	4.55|nonceps|Robust|23|LR|-	6.11|GFCC, nonceps|Robust|68|LR|SMOTE|STD	0.00|iMFCC, nonceps|MinMax|68|LR|SMOTE|PCA	0.00|nonceps|Robust|23|LR|SMOTE
Male	a	0.00|LFCC, nonceps|MinMax|15|SVM|SMOTE|SUM, STD	0.00|nonceps|Robust|16|LR|SMOTE	0.00|nonceps|Robust|31|LR|-	0.00|nonceps|Standard|16|LR|SMOTE
	i	0.00|CQCC, MFCC, BFCC|Robust|720|LR|SMOTE|PCA, SUM, STD, SKEW	0.00|MFCC, BFCC|Robust|120|RF|SMOTE|STD	0.00|CQCC, LFCC|Standard|240|LR|SMOTE|STD, SKEW	0.00|CQCC, BFCC|Unscaled|180|RF|SMOTE|PCA, SUM
	u	0.00|CQCC, LFCC|Robust|24|SVM|SMOTE|SUM, STD	0.00|NGCC, nonceps|Unscaled|46|RF|SMOTE|SKEW	0.00|iMFCC|Robust|30|LR|SMOTE|PCA	0.00|CQCC, MFCC|Standard|480|LR|SMOTE|PCA, SUM, STD, SKEW

## Data Availability

The data containing the results presented in this study are openly available in Zenodo at 10.5281/zenodo.7897603 (accessed on 22 May 2023), reference number [105]. The considered voice database is a third party data that can be obtained from: https://stimmdatenbank.coli.uni-saarland.de/help_en.php4 (accessed on 22 May 2023).

## Abbreviations

The following abbreviations are used in this manuscript:

ACC	Accuracy
ANN	Artificial Neural Network
APQ	Amplitude Perturbation Quotient
AVPD	Arabic Voice Pathology Database
BFCC	Bark-Frequency Cepstral Coefficients
CC	Cepstral Coefficient
CNN	Convolutional Neural Network
CQCC	Constant Q Cepstral Coefficient
DFT	Discrete Fourier Transform
DNN	Deep Neural Network
DPV	Database of Pathological Voices
DTCWT	Dual-Tree Complex Wavelet Transform
DWT	Discrete Wavelet Transform
EER	Equal Error Rate
EH	Error in Healthy
EMD	Empirical Mode Decomposition
EP	Error in Pathological
ETO	Energy Teager Operator
$F_{0}$	Fundamental Frequency
F1	F1-score
FFT	Fast Fourier Transform
FNN	Feedforward Neural Network
FVTL	Fitch Virtual Tract Length
GA	Genetic Algorithm
GFCC	Gammatone-Frequency Cepstral Coefficients
GMM	Gaussian Mixture Model
HNR	Harmonic-to-Noise Ratio
HOS	High-Order Statistics
ILPF	Inverse Linear Predictive Filter
iMFCC	Inverse Mel-Frequency Cepstral Coefficients
KFCV	K-Fold Cross Validation
KNN	k-Nearest Neighbors
LDA	Linear Discriminant Analysis
LFCC	Linear-Frequency Cepstral Coefficients
LPCC	Linear-Prediction Cepstral Coefficients
LR	Logistic Regression
LVQ	Learning Vector Quantization
MEEI	Massachusetts Eye and Ear Infirmary
MFCC	Mel-Frequency Cepstral Coefficients
MLP	Multilayer Perceptron
NB	Naive Bayes
NGCC	Normalized Gammachirp Cepstral Coefficients
NHR	Noise-to-Harmonic Ratio
PCA	Principal Component Analysis
PdA	Príncipe de Asturias Database
PDE	Paraconsistent Discrimination Engineering
RAP	Relative Average Perturbation
RF	Random Forest
SGD	Stochastic Gradient Descent
SMOTE	Synthetic Minority Oversampling Technique
STD	Standard Deviation
SVD	Saarbruecken Voice Database
SVM	Support Vector Machine
SWT	Stationary Wavelet Transform
UPM	Universidad Politécnica de Madrid
USPD	São Paulo University Voice Database
VTL	Virtual Tract Length
ZCR	Zero Crossing Rate
